# Precursor-dependent photoluminescence diversity in carbonized polymer dots: the luminophores and crosslinking

**DOI:** 10.1093/nsr/nwaf377

**Published:** 2025-09-09

**Authors:** Songyuan Tao, Chengyu Zheng, Bai Yang

**Affiliations:** State Key Laboratory of Supramolecular Structure and Materials, Department of Polymer Science, College of Chemistry, Jilin University, Changchun 130012, China; State Key Laboratory of Supramolecular Structure and Materials, Department of Polymer Science, College of Chemistry, Jilin University, Changchun 130012, China; State Key Laboratory of Supramolecular Structure and Materials, Department of Polymer Science, College of Chemistry, Jilin University, Changchun 130012, China

**Keywords:** carbon dots, carbonized polymer dots, photoluminescence, crosslink-enhanced emission effect, molecular-state luminophores

## Abstract

Carbonized polymer dots (CPDs) represent a prominent subclass of luminescent carbon dots (CDs), distinguished by their unique core-shell nanostructures and exceptional optical performance. Over the past decade, CPDs have experienced significant growth, as they provide a promising approach to fabricate efficient luminophores at the nanoscale through simple and environmentally friendly methods. Various precursors, including small molecules, polymers and biomass, have proved to be effective for the synthesis of CPDs, leading to a diversity of photoluminescence (PL) properties, albeit with ongoing debates regarding the origins of PL. Herein, the precursor-dependent luminophores and crosslinking reactions are expounded in several representative CPDs to reveal the enhanced PL emissive behaviors that differ from traditional small molecules or polymers. We emphasize the synergistic contributions of molecular-state fluorophores and crosslink-enhanced emission effect in highly luminous CPDs. Additionally, we propose future development trends for CD materials to foster open discussion and guidance.

## Teaser

Tailoring precursors customizes luminophores and crosslinking in nano-confined domains, yielding carbonized polymer dots with enhanced and diverse photoluminescence.

## INTRODUCTION

Carbon can form the basic skeleton of organisms, serving as a most intensively exploited element for research and application purposes. Considerable efforts have been devoted to developing carbon nanotechnology, in order to open the bandgap of macro-scaled carbon materials and regulate their electronic behaviors. Studies on carbon materials, e.g. graphite, diamond, fullerene, carbon fiber and carbon nanotube, have focused on their extraordinary mechanical, thermal and electronic transmission performance during the whole of the 1900s [1–4]. Accordingly, carbon materials were once considered to be non-luminous for a long time.

Carbon dots (CDs) were identified as luminescent carbon nanoparticles with characteristic diameters of <10 nm after their serendipitous discovery in 2004 [5]. Initially, CDs were synthesized via top-down methods by cutting *sp^2^*-hybridized carbon materials to nanoscale. Despite undergoing complex post-modification processes, the photoluminescence (PL) performance of these CDs remained unsatisfactory, i.e. low PL quantum yield (QY < 10%), short emission wavelength (λ_em_ < 550 nm) and poor water solubility [6,7].

Since the early 2010s, advancements in bottom-up synthetic methodologies have propelled the field of CDs forward at an impressive pace, particularly with the emergence of carbonized polymer dots (CPDs) [8]. A variety of precursors, including molecules, polymers and biomass, have been employed to synthesize CPDs through straightforward crosslinking and carbonization processes [9]. CPDs feature a distinct ‘core-shell’ structure, comprising an *sp^2^*/*sp^3^*-hybridized carbon core surrounded by polymer chains [10]. CPDs exhibit superior performance to traditional CDs, thence recognized as one of the most promising nanomaterials for applications in luminescence, optoelectronics, biology and energy-related fields [11–13]. The proliferation of CPDs has resulted in a rich diversity of structures and PL properties, but also confusion regarding their underlying mechanisms. Understanding what governs the PL of CPDs is vital, as it informs the effective evaluation and customization of these novel luminescent nanomaterials.

The primary aim of this review is to provide a brand-new perspective on luminophores and crosslinking to elucidate the precursor-dependent PL behaviors of CPDs (Fig. S1). In the ‘Evolution of CDs: from fluorescent carbons to CPDs’ section, we discuss the evolution of synthetic methodologies for CDs, tracing the transition from initial fluorescent carbon to current CPDs. The ‘Unique mechanisms of CPDs: formation and luminescence’ section introduces the unique formation and PL mechanisms of CPDs, highlighting the synergistic contributions of molecular-state fluorophores and crosslinked polymer structures to their enhanced PL emission. In the ‘Precursor-dependent PL diversity in CPDs: reactions and structures’ section, we exemplify the precursor-dependent PL diversity of CPDs, and illustrate the characteristic reactions of luminophores, along with their distinct PL behaviors, in several representative categories. Finally, we conclude by offering considerations on the current status and future trends of CPDs for open discussion and possible guidance. Our desire is that this review clarifies fundamental issues regarding the structure and properties of CPDs, and inspires tailored synthetic designs for CD materials.

## EVOLUTION OF CDS: FROM FLUORESCENT CARBON TO CPDS

**Figure 1.**
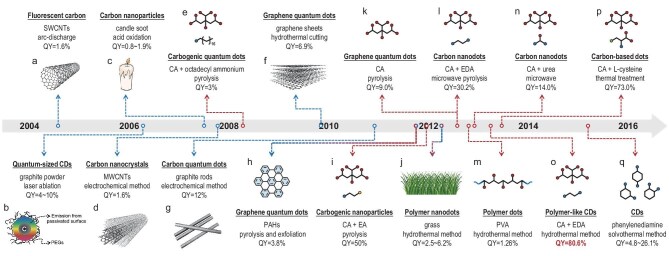
Evolution of CDs, illustrating raw materials, preparation methods, PL properties and definitions. (a) ref.[5], (b) ref.[14], (c) ref.[15], (d) ref.[16], (e) ref.[19], (f) ref.[17], (g) ref.[18], (h) ref.[20], (i) ref.[21], (j) ref.[22], (k) ref.[23], (l) ref.[24], (m) ref.[25], (n) ref.[26], (o) ref.[8], (p) ref.[27] and (q) ref.[28]. Dotted lines are color-coded to represent different synthesis methods. Blue indicates top-down synthesis, while red indicates bottom-up synthesis.

CDs were serendipitously discovered in 2004 during the purification process of single-walled carbon nanotubes (SWCNTs) from arc-discharge soot (Fig. 1a) [5]. The isolated components were initially termed ‘fluorescent carbon’, which is widely recognized as the earliest instance of CDs. Following this discovery, a variety of carbon-based precursors, such as graphite powder [14], candle soot [15], multi-walled carbon nanotubes (MWCNTs) [16], graphene sheets [17] and graphite rod [18], were utilized to synthesize luminescent nano-carbon via rigorous physical and chemical methods, e.g. laser ablation, oxidation etching, electrochemical exfoliation and hydrothermal cutting (Fig. 1a–d, f and g).

**Scheme 1.**
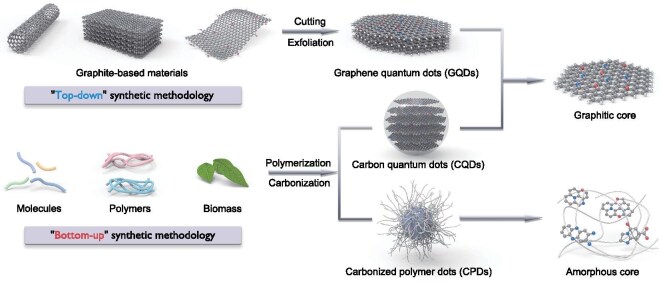
The classification of CDs as GQDs, CQDs and CPDs from the perspective of synthetic procedure and structural characteristics.

Later-developed synthetic methodologies for CDs classify the manufacturing processes that involve breaking down the bulk carbon into smaller fragments as a ‘top-down’ approach (Scheme 1). The resulting CDs inherit the structural characteristics of their carbonaceous precursors, typically comprising single or a limited number of graphene layers with functional groups attached to the surface or edge. The properties of these CDs depend on the degree of exfoliation and oxidation of the precursors. Harsh synthetic conditions often result in surface defects and a lack of hydrophilic groups on CDs. Therefore, surface-passivation and post-modification are critical steps for enhancing the PLQY and water solubility of ‘top-down’ CDs. Nevertheless, achieving a PLQY exceeding 10% has remained a significant challenge for most CD systems for an extended period (Fig. 1b, Fig. S2a) [29].

Multiple terms have been proposed over time to describe the obtained nanomaterials, e.g. carbon nanodots, carbon-based dots, carbon nanoparticles, carbogenic quantum dots, carbon nanocrystals and quantum-sized CDs, all reflecting their structural characteristics and elemental composition. Despite the minimal quantum effect observed, the terms graphene quantum dots (GQDs) and carbon quantum dots (CQDs) are frequently used in related research, evolving into two main subclasses of CD materials (Scheme 1). Top-down approaches dominated the synthesis of CDs until 2013, despite notable shortcomings, such as high processing costs, severe operational conditions and unsatisfactory PL performance.

Molecule precursors, i.e. citric acid (CA) and octadecyl ammonium, were first employed to fabricate CDs by pyrolysis in 2008, marking the beginning of a new synthetic era for CDs (Fig. 1e) [19]. In contrast to the previously mentioned carbonaceous precursors, molecule-derived CD systems provide more possibilities for simplified synthesis and improved performance. Precursor selections have included polycyclic aromatic hydrocarbons (PAHs), molecules or polymers containing carboxyl, amino and hydroxyl groups, and even biomass (Fig. 1e, h–q) [8,19–28]. A milestone study appeared in 2013, when Zhu *et al.* hydrothermally synthesized CDs from CA and ethylenediamine (EDA), achieving an unprecedented PLQY of up to 80% and a high product yield exceeding 50% (Fig. 1o) [8]. This breakthrough ushered in explosive growth in CD research and a series of performance enhancements (Fig. S2a). Several representative CD systems have successively been reported, including CA-EDA, CA-urea, phenylenediamine (PDA) and various aqueous polymers.

These methods for generating CDs from small molecules or macromolecules are categorized as ‘bottom-up’ methodologies (Scheme 1), already developed into pyrolysis, solvothermal or hydrothermal methods, as well as ultrasound and microwave-assisted techniques. In addition to ease of operation, bottom-up synthesized CDs exhibit diverse structures and excellent PL performance. Until now, the PL emission spectra of reported CDs have spanned from ultraviolet (UV) to near-infrared (NIR), with many exhibiting PLQYs exceeding 90% and approaching 100% in most regions [30]. Bottom-up methods have increasingly supplanted top-down techniques, establishing themselves as the predominant synthetic methodology for photoluminescent CDs.

The term CPDs is a newly proposed definition describing a highly crosslinked carbon core with slight graphitic sub-domain structure, enveloped by an abundance of hydrophilic polymer chains (Scheme 1). The core-shell architecture of CPDs establishes them as ideal photofunctional materials. Within this structure, the crosslinked core spatially confines luminophores to reduce non-radiative transitions while enhancing excited-state coupling, thereby promoting diverse radiative pathways. Concurrently, the polymeric shell facilitates integration with other materials, enabling functional hybridization in multifunctional composites.

The majority of CDs synthesized via bottom-up routes should be categorized as CPDs due to the asymmetry of precursors and the randomness of reactions. CPD particles typically exhibit amorphous characteristics with low crystallinity, though some display lattice fringes with spacings distinct from graphite, attributed to the orderly arrangement of polymer chains. Thus, CDs can be essentially interpreted as *sp^2^*-hybridized carbon clusters isolated within *sp^3^*-hybridized carbon matrices, with their performance largely dictated by the composition and ratio of these domains.

Consistency in terminology is essential for performance comparison and targeted synthesis of CDs [31,32]. A comprehensive nomenclature has been established outlining a detailed series of naming rules with a prioritized order of various factors (Fig. S2b) [33]. This nomenclature system will facilitate understanding of the precursor-dependent structural diversity in CPDs, and potentially index the library of CPD materials following long-term development.

## UNIQUE MECHANISMS OF CPDS: FORMATION AND LUMINESCENCE

**Scheme 2.**
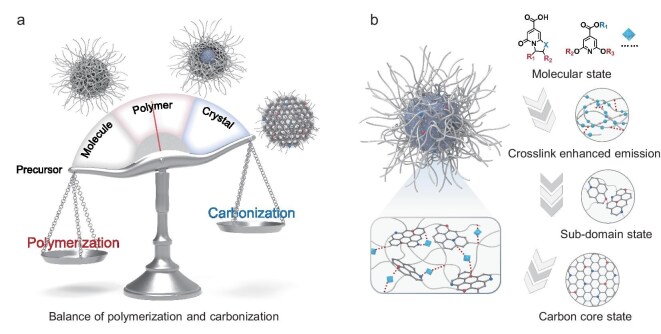
(a) The balance of carbonization and polymerization during the formation of CPDs. (b) The structure of CPDs and the dominant PL origin at different growth stages.

The uniqueness and advantages of CPDs arise from their distinctive formation and PL mechanisms. Molecule- or polymer-derived precursors of CPDs typically undergo hydrothermal crosslinking. The elevated temperature and pressure conditions during hydrothermal synthesis can activate new reaction pathways, generating specialized fluorophores embedded within crosslinked networks to form nanoparticles. The molecular-state luminophores and crosslink-enhanced emission (CEE) effect are the primary origins of PL in CPDs, contributing to enhanced performance in terms of PLQY and stability. The structural and functional attributes of CPDs are predominantly regulated by the balance between polymerization and carbonization (Scheme 2a) [11].

### The growth of CPDs: from precursors to luminous nanoparticles

As illustrated in Scheme 2b, bright organic fluorophores are generated by intramolecular cyclization during the initial stages of the reaction, remaining free in the solvent. As intermolecular dehydration and crosslinking increase, polymer chains or clusters form, with dissociative fluorophores acting as molecular states being grafted onto or embedded within the polymeric backbone. The resulting crosslinked polymer networks provide a stable chemical environment for these molecular-state fluorophores, enhancing intraparticle interactions and thereby improving PL emission. As the reaction proceeds, reversible dehydration transitions to irreversible carbonization, including reactions such as dehydrogenation and deamination. Some fluorophores and the surrounding polymer chains may subsequently carbonize into heteroatom-doped graphitic fragments, referred to as the sub-domain state. Furthermore, the carbon core state encompasses structures with extensive conjugated π-domains that form as the terminal state after complete carbonization [34,35].

The luminescent behavior of CPDs is critically governed by the interplay between crosslinking degree and carbonization extent. Enhanced crosslinking restricts molecular motion to improve PLQY, but may cause aggregation-caused quenching if excessive. Conversely, deeper carbonization expands *sp^2^*-conjugated domains, inducing emission red-shifts, whereas incomplete or over-carbonization either retains unstable fluorophores or generates slightly luminous graphitic structures [36,37]. Consequently, achieving optimal PLQY and color tunability requires balancing these parameters: crosslinking confines emissive centers spatially, while controlled carbonization modulates the emissive bandgap energy.

Notably, complete graphitization is typically achieved at 2300°C–3000°C, and common bottom-up techniques for CPD synthesis are far insufficient to achieve a true carbon core state [38]. The previously reported PL attributed to the carbon core state should instead be assigned to molecular or sub-domain states. Moreover, attaining a genuine carbon core state is often unnecessary due to its lower PLQY compared to other states, unless for higher pursuits in conductivity or stability. Recent reports have introduced the concepts of ‘crosslinking-induced nucleation and carbonization’ [39] and ‘crosslinking synergistically inducing quantum-state luminescence’ [12], offering new insights into the unique formation and luminescence mechanisms of CPDs. Crosslinking undertakes a crucial role in generating the luminous structures and regulating their interactions.

### The dominant PL origin of CPDs: luminophores and crosslinking

#### Molecular state originating from organic luminophore

Molecular states are typically interpreted as strongly emissive organic fluorophores or specific aromatic segments attached to the surface or embedded within the carbon skeleton of CPDs. The PL performance of the molecular state largely depends on the structure of the molecular precursors and the extent of the reactions involved.

**Figure 2.**
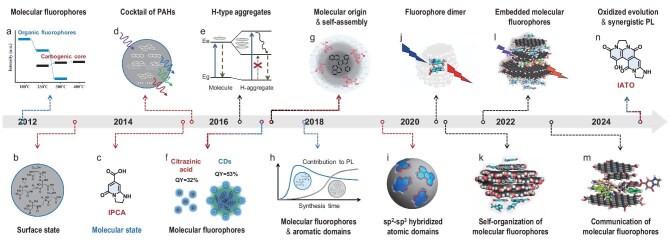
Development of the molecular state in CPDs. (a) ref.[21], (b) ref.[8], (c) ref.[40], (d) ref.[41], (e) ref.[42], (f) ref.[43], (g) ref.[44], (h) ref.[45], (i) ref.[46], (j) ref.[47], (k) ref.[48], (l) ref.[49], (m) ref.[50] and (n) ref.[51]. The dotted lines are color-coded to represent three main aspects of molecular fluorophores. Blue indicates evolution, red indicates structure and black indicates interaction.

Molecular fluorophores dominate the PL of CPDs at low synthetic temperature [21] (Fig. 2a) or the initial stage of formation [45] (Fig. 2h). Carbonization occurs as the temperature increases or the reaction progresses, which brings about the extension and fusion of molecular fluorophores into larger aromatic domains. Surface-state emission, akin to the molecular state, was initially mentioned to describe the distinct PL behaviors arising from the quantum size effect of defect-free carbon core (Fig. 2b) [8]. A cocktail model of PAHs was proposed to illustrate the structure of CPDs (Fig. 2d), in which PAHs are embedded in *sp^3^*-hybridized carbon matrix [41]. A quantum chemical approach further optimized the model of CPDs, depicting *sp^2^*-hybridized carbon sub-domains situated within the *sp^3^*-hybridized amorphous core (Fig. 2i) and highlighting the significance of *sp^2^-sp^3^* hybridized atomic domains in the optical properties of CPDs [46].

In 2015, a bright-blue fluorophore, denoted as IPCA (5-oxo-1,2,3,5-tetrahydroimidazo[1,2-a]pyridine-7-carboxylic acid, Fig. 2c), with a specific molecular structure from CA-EDA CPDs, was first confirmed via column chromatography separation [40]. CPDs exhibit superior photostability and water solubility to that of individual molecules. Recently, another green fluorophore, termed IATO (3-hydroxy-7,8,13,14-tetrahaydroimidazo-[2,3-k][4,7,11]-triazaacephenanthrylene-1,5,10(12H)-trione, Fig. 2n) was identified in CA-EDA CPDs by a reprecipitation method [51]. These findings revealed an oxidized evolution from IPCA to IATO, and elucidated the synergistic PL of multiple molecular fluorophores. It is remarkable that the discrepant PL behaviors confirmed molecular fluorophores to be attached to the CPDs instead of just free in solution (Fig. 2f) [43]. The contribution of the molecular state to the PL in CPDs is substantially greater than that of the *sp²*-hybridized core in various sizes or the surface-related states.

Aggregation and interaction of molecular fluorophores are crucial for the unique emissive behaviors observed in CPDs. Single-particle measurements demonstrated that CPDs were the assemblies of surface-exposed fluorophores (Fig. 2e) [42]. The optical properties originated from H-aggregate-type excitonic states, with coherence spreading over the whole particle. H-aggregates formed through different self-assembly modes generating multiple discrete electronic states of CPDs (Fig. 2g) [44]. Moreover, evidence from the observed spectral splitting [44] (Fig. 2g) and the calculated optical properties [47–50] (Fig. 2j–m) supported the coupling of molecular fluorophores in CPDs, both experimentally and theoretically. Consequently, CPDs exhibit increased variability in PL performance due to these precursor-dependent fluorophores and their adjustable interactions.

#### CEE effect originating from crosslinked polymer structure

Crosslinking serves as the forming base of nano-structure that is prevalent in CPDs. The CEE effect emphasizes the contribution of crosslinking to PL and reflects the polymeric characteristics of CPDs. It is deemed to be a unique mechanism for CPDs, and also an important complement to the molecular-state PL dominated by conjugated luminophores with a well-defined structure.

**Figure 3.**
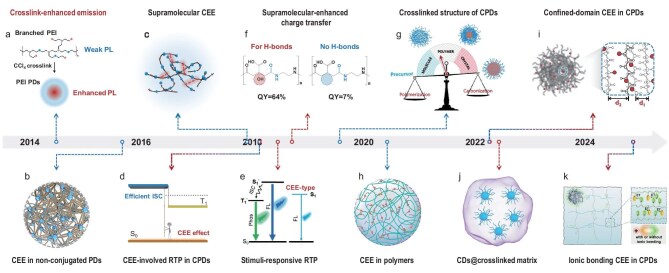
Development of the CEE effect: (a) ref.[52], (b) ref.[53], (c) ref.[54], (d) ref.[55], (e) ref.[56], (f) ref.[57], (g) ref.[11], (h) ref.[58], (i) ref.[59], (j) ref.[60] and (k) ref.[61]. Dotted lines are color-coded to represent three main aspects of the molecular fluorophores. Blue indicates mechanism, and black indicates performance.

The pioneering concept of the CEE effect was first put forward in 2014. A crosslinking model based on polyethyleneimine (PEI) was established to evaluate the enhanced PL behaviors initiated by the CEE effect in CPDs (Fig. 3a, Fig. S3a) [52]. Chemical crosslinking or physical immobilization of polymer chains was recognized as critical in eliciting the unconventional PL emission from sub-luminophores within non-conjugated polymer dots containing electron-rich heteroatom double bonds (e.g. C=O, C=N, N=O, P=O) or single bonds (e.g. C–O, C–N, N–P) (Fig. 3b) [53]. Furthermore, the CEE effect also exerts its effect in polymer-related systems by suppressing non-radiative de-excitation and promoting radiative transition (Fig. S3b) [58]. It involves not only several forms of bonding interactions, i.e. covalent-bond CEE [52,55] (Fig. 3a and d), supramolecular-interaction CEE [54] (Fig. 3c), ionic-bonding CEE [58,61] (Fig. 3h and k), but also the non-bonding interactions, i.e. confined-domain CEE [59] (Fig. 3i). These interactions increase the electron-cloud coupling and energy level splitting within one particle (Fig. 3g), resulting in additional emissions from CPDs that differ from those of individual luminophores [11].

Density functional theory (DFT) calculations were performed on CA-EDA CPDs (Fig. 3f) [57]. The results attributed the origin of the universal blue fluorescence of CPDs to photo-induced charge transfer between functional groups within entangled polyamide chains, while enhanced by intermolecular hydrogen-bonding mediated by supramolecular interactions. Apart from fluorescence, the CEE effect also plays a crucial role in phosphorescence. A self-protective strategy inspired by the CEE effect was proposed to utilize the crosslinked structure of CPDs as an effective matrix for protecting triplet excitons, thus first achieving metal-free room temperature phosphorescence (RTP) of CD-class materials without matrix-assisting (Fig. 3d) [55]. Since high temperature can facilitate the dehydration and carbonization of intertwined polymer chains to form a dense core in CPDs, a stimuli-responsive PL with a conversion from fluorescence to ultralong RTP was observed in CPDs by an external heating stimulus (Fig. 3e) [56]. Recently, significant RTP phenomena have been reported in CEE-type CPDs, e.g. time-dependent RTP by modulating intraparticle hydrogen bonds or ionic bonding [61] (Fig. 3k), and NIR RTP by designing CEE-related precursors [62]. Besides, research exploring ‘CDs@crosslinked matrix’ indicates that adjusting the crosslinking strength between CPDs and matrices can dramatically enhance the RTP properties of composite materials (Fig. 3j) [60]. The CEE effect promotes the emission from non-luminous to luminous, weakly luminous to strongly luminous, and even modifies the mode of radiative transition.

The synergistic contributions of molecular-state luminophores and crosslinked polymer structures are unique and significant to the enhanced PL emission in CPDs. Firstly, the molecular state is the primary source of strong PL in CPDs. Secondly, the CEE is ubiquitous since crosslinking is a prerequisite for the growth of CPD particles. Thirdly, the molecular state and the CEE effect form the structural basis for further carbonization, thereby determining the types and sites of element doping in the sub-domain state and the carbon core state.

## PRECURSOR-DEPENDENT PL DIVERSITY IN CPDS: REACTIONS AND STRUCTURES

This section exemplifies precursor-dependent PL behaviors in several representative systems of CPDs, including non-aromatic molecule precursors (e.g. CA-EDA CPDs and CA-urea CPDs), aromatic molecule precursors (e.g. aminobenzene-based CPDs and hydroxybenzene-based CPDs) and polymer precursors (e.g. polymer-derived CPDs synthesized via polycondensation or addition polymerization). The discussions involve the forming reactions of molecular-state luminophores and their crosslinked structures. Meanwhile, the multifarious properties of CPDs derived from similar precursors will be sorted out to demonstrate the diversity in precursor-dependent PL.

### Non-aromatic molecule precursor-derived CPDs

CA serves as one of the most extensively studied non-aromatic precursors for CPDs that are inherently non-luminescent. As a quadrifunctional tricarboxylic acid, CA can easily dehydrate or decarbonylate upon heating, leading to the formation of extended conjugated π-domains. Meanwhile, CA can readily react with amines or alcohols to generate cyclic molecular compounds driven by structural stress, alongside the formation of polyamides and polyesters. CA-derived CPDs, synthesized from single-component or multi-component precursors, exhibit both high reactivity and distinct PL performance.

#### CA and EDA

**Figure 4.**
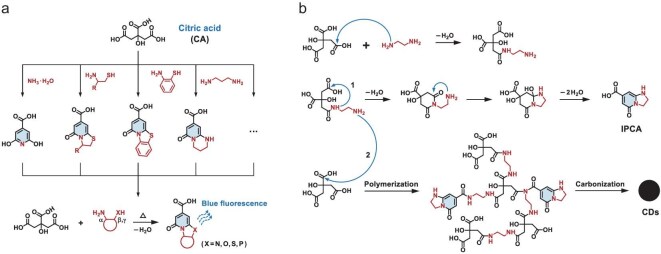
(a) Formation of fluorescent 2-pyridones from CA and amines. (b) Possible reaction process during the formation of CA–EDA CPDs.

The earliest report on the PL of CA dates back to the 19th century. In 1892, Easterfield *et al.* first elaborated that CA could form blue-emitting citrazinic acid by thermal condensation with ammonia (Fig. 4a) [63]. In the 1970s, Olthoff and Hori successively investigated the PL behaviors of highly fluorescent ring-fused dihydrothiazolo 2-pyridone derivatives produced from CA and aliphatic or aromatic β-amino thiols (Fig. 4a) [64,65]. Kasprzyk *et al.* elucidated the chemical structures of similar fluorophores in photoluminescent polymers in 2013 [66] and proposed a general access to synthesizing numerous derivatives of ring-fused 2-pyridones via thermal condensation of CA with β-substituted or γ-substituted amines in 2015 [67] (Fig. 4a, Table S1). Although these fluorophores were created via organic synthesis, defining their structures significantly aids in understanding the molecular-state PL of CA-derived CPDs.

CA is the first molecular precursor utilized in the synthesis of CPDs. The PL origins of CA-derived CPDs have been extensively explored since CA-EDA CPDs marked a significant breakthrough in PLQY in 2013 [8]. Song *et al.* purified the IPCA molecule via column chromatography and confirmed the co-existence of IPCA, polymer clusters and carbon nanoparticles [40]. IPCA was believed to be an independent fluorophore at the initial stage, and possibly bonded to the surface or internal core by covalent crosslinking as the reaction proceeded (Fig. 4b, Table S1) [68]. Xiong *et al.* summarized the synthetic pathways leading to the formation of molecular fluorophores in CA-derived CPDs, indicating that PL performance was a complex interaction of stand-alone molecular fluorophores and their aggregates embedded within an amorphous carbon/polymer network [69].

Duan *et al.* identified and quantified the IPCA fluorophore in CA-EDA CPDs by multi-nuclear solid-state nuclear magnetic resonance (NMR) (Fig. S4a) [70]. The results demonstrated that IPCA was finely dispersed in a polyamide matrix, with the transformation of carboxyl groups of IPCA through amide or ester formation. Langer *et al.* performed quantum mechanics/molecular mechanics calculations to investigate changes in the PL behaviors of molecular-state IPCA fluorophores within structural models of CPDs (Fig. 2l, Fig. S4b) [49]. They found the environment of IPCA and its embedded forms markedly influenced the PL intensity and excitation–emission Stokes shifts of CPDs. Moreover, they examined the conformational behaviors and optical properties of IPCA dimer as PL centers in CPDs [47] (Fig. 2j), illustrating the spontaneous stacking of IPCA in aqueous solution into CPD structures [48] (Fig. 2k), and the communication of molecular fluorophores with other PL centers by systematic theoretical simulations [50] (Fig. 2m).

Table S1 summarizes the molecular-state fluorophores and their corresponding properties of CA–amine CPDs, excluding organic solvothermal synthesis. The following conclusions can be drawn about the molecular-state fluorophores of CA–amine CPDs: (i) it originates from ring-fused 2-pyridone derivatives, with CA constituting a basic six-membered skeleton of 2-pyridone with amine determining the structure of the fused ring; (ii) it emits within the blue spectral range with extremely high PLQY, exhibiting similar PL properties to isolated molecules but is affected by aggregation behavior; (iii) low temperature, i.e. <160^o^C, promotes the formation of the molecular state, while high temperature can disrupt these fluorophores for fusion growth.

#### CA and urea

**Figure 5.**
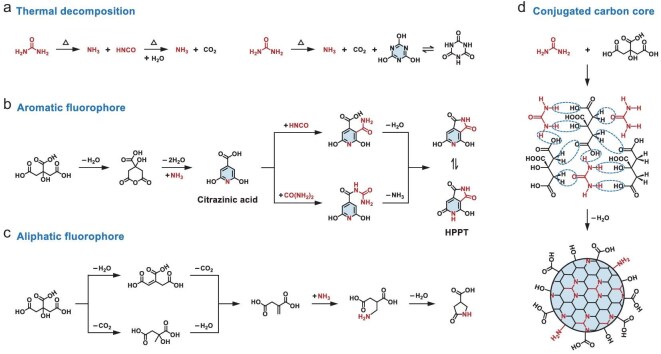
Possible reaction processes during the formation of CA–urea CPDs, including (a) thermal decomposition of raw materials, the generation of (b) aromatic or (c) aliphatic fluorophores and (d) conjugated carbon core.

As previously mentioned, the reactions forming 2-pyridone-derived fluorophores predominantly dictate the growth process of CA–amine CPDs, owing to the strong nucleophilicity of amino groups and the high thermal stability of amine precursors, thereby avoiding the occurrence of numerous side reactions. The molecular state of CA–amine CPDs predominantly occupies the blue-emissive PL regions, with emission wavelengths being challenging to tune despite exceptional PLQY. Researchers have long sought methods to achieve full-color PL in CPDs from non-aromatic precursors, particularly in long-wavelength emission regions, until the comprehensive investigation of CA–urea CPDs provided new insights. Owing to the comparable reactivity and matched configurations of precursors, CA–urea CPDs hold multiple reaction pathways, e.g. thermal decomposition (Fig. 5a), formation of molecular-state fluorophore (Fig. 5b and c) or conjugated carbon core (Fig. 5d), thus leading to diverse PL properties.

In terms of precursor molecules, either CA or urea can undergo thermal decomposition and structural transformation, the products of which probably participate in subsequent reactions. Urea typically converts to isocyanic acid and ammonia by pyrolysis of single or multiple molecules [71] (Fig. 5a), while CA tends to undergo intramolecular cyclization and aromatization via dehydration [63] (Fig. 5b). Notably, the effect of solvents must also be considered, even for single-component precursors, as solvents can act as carriers and react with the precursors (e.g. CA–CPDs, Table S2).

In terms of the molecular state, two types of fluorophores have been confirmed, respectively consisting of aromatic and aliphatic structures. Kasprzyk *et al.* elucidated the chemical structure of the aromatic fluorophore 4-hydroxy-1H-pyrrolo[3,4-c]pyridine-1,3,6(2H,5H)-trione (HPPT) responsible for the green PL of CA–urea CPDs by microwave-assisted pyrolysis in open-vessel reactors [72]. The molecular-state fluorophore of HPPT can be formed via two possible pathways under water-free conditions (Fig. 5b), but supplanted by blue-emissive citrazinic acid in an aqueous environment. Interestingly, Yao *et al.* discovered a novel blue-emissive aliphatic fluorophore of CA–urea CPDs by microwave-assisted hydrothermal treatment, named 5-oxopyrrolidine-3-carboxylic acid (Fig. 5c) [73]. This fluorophore exhibits aggregation-enhanced emission, unlike the typical aggregation-caused quenching observed in other molecular states of CPDs. The PL behaviors were supposed to be associated with the crosslinked polymeric skeleton within CPDs, largely dependent on the interactions with the polymers or molecular-state fluorophores.

In terms of the carbon core, CA can self-assemble into a sheet structure under basic conditions, forming a graphite framework resembling oxygen-containing GQDs via intermolecular dehydration. Concurrently, CA and urea can dehydrate into well-defined π-conjugated cores with rich nitrogen doping, attributed to their highly complementary molecular configurations (Fig. 5d). Consequently, CA–urea CPDs can more readily form regular carbon core by structure-driven dehydration than other CPDs that depend on harsher dehydrogenation or deamination processes. Liu *et al.* developed a selective preparation from GQDs to graphene oxide (GO) by tuning the carbonization degree of CA [22]. CA could be partially carbonized to GQDs comprising abundant isolated *sp^2^* clusters (PLQY = 9.0%), or fully carbonized to larger nanosheets, i.e. GO (PLQY = 2.2%). Qu *et al.* designed a series of CA–urea CPDs to investigate the nitrogen-doping process through a hydrothermal route (Table S2) [74]. The introduction of nitrogen into the graphene framework led to substantial improvements in PLQY, which occurred during the formation of the pyrrolic nitrogen by dehydration between amide and carboxyl groups (PLQY = 58%) and subsequently the conversion of pyrrolic nitrogen into graphite nitrogen (PLQY = 78%). Tian *et al.* controlled the bandgap emission of CA–urea CPDs by solvothermal synthesis (Table S2) [75]. The selected solvents, i.e. water, glycerol and dimethyl formamide (DMF), governed the degree of dehydration and carbonization of precursors, leading to the formation of extended *sp^2^*-conjugated domains within CA–urea CPDs and tunable wavelength ranging from blue- to red-emitting regions.

Theoretical calculations provide effective insights into the PL behaviors arising from carbonized domains, which are often difficult to characterize. Sk *et al.* employed time-dependent DFT (TDDFT) methods to calculate the emission wavelengths of ideal zigzag-edged GQDs models of varying diameters [76]. The observed linear correlation (Fig. S5a) indicated a size-dependent PL performance of GQDs, with emissions spanning the entire visible region (400–700 nm) as the diameter varied from 0.89 to 1.80 nm. Holá *et al.* evaluated the electronic and optical properties of pyrene-based models to elucidate the influence of nitrogen doping in CPDs (Table S2, Fig. S5b) [77]. Their calculations suggested that the presence of graphitic nitrogen created mid-gap states in the original highest occupied molecular orbit (HOMO)–lowest unoccupied molecular orbit (LUMO) gap of undoped systems, thereby resulting in the narrowing of the bandgap and the red-shifting in absorption and PL peaks. Furthermore, Cao *et al.* explored the relationship of charge transfer and PL performance in amino-functionalized CPDs by a similar computational approach, which highlighted the significance of nucleophilic substitution positions for achieving long-wavelength red emissions [78].

Miao *et al.* changed the solvothermal-pyrolysis temperature and precursor ratios to regulate the graphitization and surface functionalization of CA–urea CPDs (Table S2, Fig. S5c), achieving tunable PL emissions from 430 to 630 nm [79]. Accordingly, apart from molecular fluorophores, the PL performance of CA–urea CPDs can be affected by the structural characteristics of carbonized domains after further graphitization, e.g. conjugated dimension, heteroatom doping and substituent groups [37,80].

### Aromatic molecule precursor-derived CPDs

Aromatic precursors significantly contribute to the construction of strongly emissive CPDs, particularly in the long-wavelength regions, due to the inherent advantages of conjugated structures in PL [81]. The planarization of aromatic rings promotes orientation during CPD growth, resulting in regular morphology and regulated PL performance. This section focuses on two categories of aromatic precursors used for CPD synthesis: aminobenzene-based derivatives and hydroxybenzene-based derivatives.

#### Aminobenzene-based derivatives

**Figure 6.**
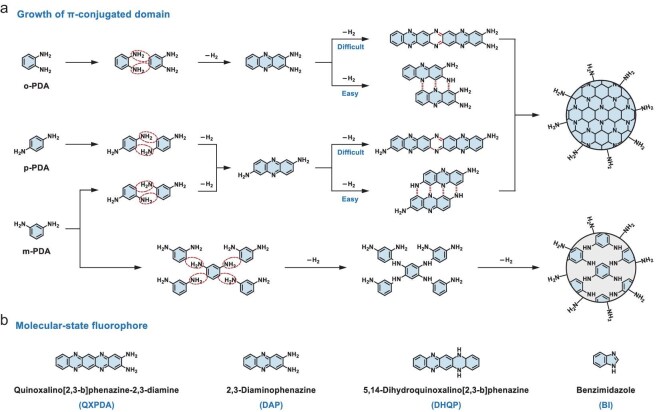
(a) Possible reaction processes during the formation of CPDs synthesized from *o, p* and *m*-PDA. (b) Confirmed molecular-state fluorophores.

In the early 2010s, it proved challenging to shift from blue/green emission of CPDs to red-shifted PL. In 2015, Jiang *et al.* reported an easy solvothermal approach for synthesizing red-, green- and blue-emissive CPDs using three isomers of PDA as precursors, i.e. *o*-PDA, *m*-PDA and *p*-PDA (Fig. 6a) [28]. This work initiated another flourishing period of research into CPDs, focusing on various aminobenzene-based precursors (Table S3, Table S4). Significant advancements have since been made regarding the growth processes and molecular-state fluorophores of aminobenzene-derived CPDs (Fig. 6).

Regarding the growth of π-conjugated domains, CPD particles can grow along the horizontal or vertical direction, influenced by the dehydrogenation reactions occurring at differently substituted sites. Back in 2001, Thomas *et al.* investigated the electrochemical polymerization of *o*-PDA dimer (DAP), also noted in the polymerization of *o*-PDA [82]. The calculated energies indicated that these polymers predominantly consisted of poly(aminophenazines), mainly comprising phenazine repeat units with minimal extended ladder segments. Yan *et al.* demonstrated the quantum size effect in amorphous *o*-PDA CPDs synthesized via hydrothermal treatment. The MALDI-TOF mass spectra and theoretical calculations suggested that *o*-PDA CPDs of varying sizes retained the same chemical component (oligomers, such as dimers, trimers and tetramers) but exhibited larger bandgaps due to quantum size effect (Fig. S6b, Table S3) [83]. Yang *et al.* fabricated 2D C_3_N crystals from DAP through hydrothermal methods at high temperatures, resembling nitrogen-doped GQDs (Table S4) [84]. Theoretical analyses suggested that the vertical polymerization forming C–N bonds was thermodynamically more favorable than horizontal polymerization or N–N bond formation (Fig. 6a). These growth principles were corroborated by other research groups, including Tan *et al*. [85] and Wang *et al*. [86]. Additionally, the substitution sites of amino groups render *o*-PDA and *p*-PDA CPDs more favorable for forming extended *sp^2^*-conjugated domains, independent of solvent influence, potentially leading to long-wavelength PL emissions, compared to *m*-PDA CPDs (Fig. 6a, Table S3).

In terms of the molecular state, several phenazine-based fluorophores have been successively identified, e.g. quinoxalino[2,3-b]phenazine-2,3-diamine (QXPDA), DAP, 5,14-dihydroquinoxalino[2,3-b]phenazine (DHQP) and benzimidazole (BI), which contribute to the PL emission in aminobenzene-derived CPDs (Fig. 6b). Soni *et al.* confirmed the presence of a significant amount of a molecular fluorophore within *o*-PDA CPDs (Fig. 6b, Table S3), designating the separated red-emissive component as QXPDA [87]. The blue-emissive component, exhibiting increased PLQY, was attributed to the nitrogen-doped carbon core and the aggregation-induced rigidity of the molecular state. Zhang *et al.* proposed that the PL of *o*-PDA CPDs was primarily determined by the DAP fluorophore and its interaction with the carbon framework (Fig. S6a, Table S3) [88]. The edge amino protonation of DAP significantly altered the molecular state, lowering the bandgap for photon transitions and triggering high-color-purity red fluorescence emissions via one-photon and NIR-induced two-photon mechanisms. Li *et al.* explored the formation and PL mechanism of red-emissive *o*-dihydroxybenzene (DHB)–*o*-PDA CPDs, attributing the PL to the DHQP fluorophore (Fig. 6b, Table S4) [89]. As the reaction proceeded, fluorescent DHQP molecules could be connected at the edge of CPDs and ultimately integrated into the conjugated CPD core by *sp^3^*-hybridization. Recently, they produced three-color emissive CPDs using only *o*-PDA by modulating the ratios of ethanol and DMF [90]. BI, DAP and DHQP were identified as the fluorescent molecular groups for blue-, green- and red-emissive CPDs by analyzing their optical properties (Fig. 6b, Table S4).

Acid catalysis appears to be particularly effective in modulating PL for aminobenzene-derived CPDs, promoting protonation or carbonization rather than simply introducing another precursor. Wang *et al.* synthesized six different *o*-PDA-derived CPDs that exhibited nearly identical PL behaviors after purification (Fig. S6c, Table S3) [86]. Structural and spectral characterizations indicated that these CPDs possessed similar carbon core structures that dominated the emission wavelength, with varying surface polymer shells affecting PL intensity. *o*-PDA-based CPDs were confirmed as unique materials combining the characteristics of organic molecules and quantum dots, producing electron–phonon coupling-assisted red PL. They later unveiled the PL mechanisms for *o*-PDA-derived CPDs through tunable NIR dual-wavelength lasing [91]. DHQP, confined to the CPD surface, acted as aggregation sites, while conjugated carbon core encouraged multiple scattering and enhanced light amplification. The whole CPD exhibited a CEE effect with higher PLQY (31.35% for DHQP; 60.62% for CPDs), more stable laser emission and lower laser threshold.

Beyond PDA isomers, aminobenzene-derived precursors show promise in important application areas, such as optoelectronic materials and biomedical science [13]. Yuan *et al.* reported the first exhibit of bright multi-color bandgap fluorescent CPDs utilizing CA and diaminonaphthalene (DAN) as precursors (Table S4) [92]. DAN played a crucial role in generating bandgap fluorescent CPDs, serving as a building block for intact *sp^2^* clusters that were nitrogen-doped in a large rigid π-conjugated structure and highly surface-passivated with amino at edge sites. DAN–CA CPDs were directly fabricated as the active emission layer in light-emitting diodes (LEDs), demonstrating the best performance for CD-based monochrome electroluminescent LEDs at the time. Subsequently, they developed efficient red bandgap emissive CPDs through the solvothermal treatment of *N,N*-dimethyl-, *N,N*-diethyl- and *N,N*-dipropyl-*p*-PDA in DMF, achieving high-performance electroluminescent warm-white LEDs (Table S4) [93]. The red bandgap PL emission was attributed to the π-conjugated carbon skeleton, with the passivation of -NMe_2_, -NEt_2_ and -NPr_2_ inducing charge transfer excited states that resulted in high PLQY and red-shifted emission wavelength. Yuan *et al.* proposed a two-step strategy to improve the surface passivation of solvothermally synthesized DAN–CA CPDs via amination treatment (Table S4) [94]. This efficient edge amination reduced spectral broadening caused by the wave-function polarization from thermal vibrations of oxygen-containing functional groups. The post-treated DAN–CA CPDs emitted high-color-purity deep-blue narrow-bandwidth PL, with device efficiency significantly surpassing those of previously reported quantum-tuned solution-processed deep-blue LEDs.

In the biological domain, Li *et al.* reported large amino acid-mimicking CPDs with paired α-carboxyl and amino groups on the edges, synthesized from 1,4,5,8-tetraminoanthraquinone (TAAQ) and CA (Table S4) [95]. TAAQ–CA CPDs could incorporate aromatic drugs via π–π stacking interactions, enabling NIR fluorescence and photoacoustic imaging of tumors without detectable toxicity, leading to a reduction in tumor burden following targeted chemotherapy delivery. Other noteworthy results related to aminobenzene-derived CPDs are listed in Tables S3 and S4.

#### Hydroxybenzene-based derivatives

**Figure 7.**
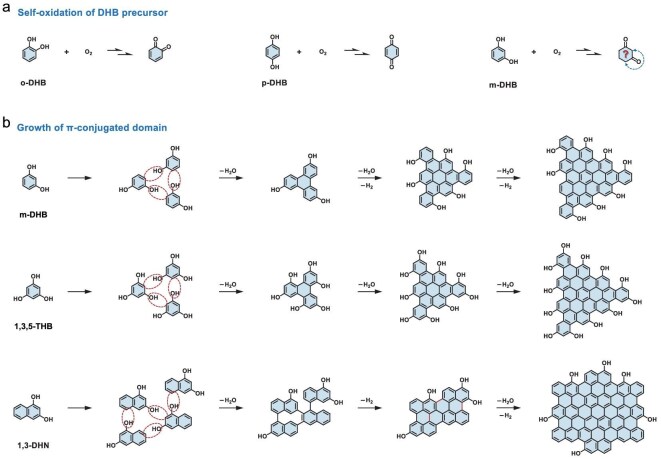
(a) Self-oxidation reaction of DHB molecules. (b) Possible reaction pathways during the formation of CPDs synthesized from DHB and its derivatives.

Hydroxybenzene-based derivatives, e.g. DHB, trihydroxybenzene (THB), dihydroxynaphthalene (DHN), represent another significant category of aromatic molecule precursors for CPD synthesis (Fig. 7). Similar to aminobenzene-derived molecules, reactions involving precursors typically result in the formation of condensed rings or *sp^2^*-conjugated domains. The molecular-state fluorophores participate in the π-electron delocalization in these conjugated domains, indicating that the effective conjugated size and edge composition are critical for the PL emission of these CPDs.

Self-oxidation of hydroxybenzene precursors is an unavoidable reaction in the synthesis of hydroxybenzene-derived CPDs [96]. Hydroxybenzene can be oxidized into quinones, e.g. *o*-DHB and *p*-DHB (Fig. 7a). However, *m*-DHB self-oxidation results in complexities, as its oxidized structure does not conform to the expected conjugated electronic configuration of benzoquinone, necessitating further exploration. Studies indicate that quinone products are unstable and rapidly polymerize through covalent attachment and aggregation under alkaline conditions [97,98]. Inspired by the self-oxidation and auto-polymerization of hydroxybenzene, Han *et al.* developed a mild and easy synthetic method based on Schiff base reactions to fabricate multi-color CPDs from *p*-DHB and EDA (Table S5) [99]. The PL emission of *p*-DHB–EDA CPDs was closely associated with the C=N functional groups on the surface. The bandgap narrowed with the increase of C=N bond content, resulting in a red-shifted PL emission. Wang *et al.* synthesized three types of CPDs with multiple emissions derived from DHB isomers (Table S5) [96]. They identified 1,2-benzoquinone and 1,4-benzoquinone as the oxidation products of *o*-DHB and *p*-DHB, respectively (Fig. 7a), which was attributed to CPD formation through dehydration, crosslinking and carbonization. Additionally, particle size and the amount of nitrogen-doping were found to collectively influence the PL emission of CPDs.

In the presence of oxidants, phenolic hydroxyl groups can be oxidized to generate the potential phenoxy radicals, which may polymerize to form polyphenols. Thus, introducing oxidants enhances the formation and PL regulation of hydroxybenzene-derived CPDs. Wang *et al.* demonstrated the successful synthesis of highly efficient red-emissive CPDs via solvothermal treatment of 1,3-DHN using potassium periodate (KIO₄) as an oxidant, achieving a record-high PLQY of 81.4% for red-fluorescent CDs (Table S5) [100]. The conjugated π domains of CPDs were expanded through a novel sequential dehydrative condensation and dehydrogenative planarization (DCDP) approach, enabling size tuning and enhancing π-conjugation (Fig. 7b). Zhu *et al.* reported an ethanothermal synthesis of multi-color fluorescent CPDs utilizing various hydroxybenzene-based derivatives and oxidants (Table S5) [101]. The oxidants oxidized phenolic hydroxyl groups and introduced abundant oxygen-containing species, e.g. carbonyl and carboxyl groups, into amorphous CPDs. The element composition of these oxidants was found to influence the PL emission wavelength of CPDs.

The growth orientation of π-conjugated domains is another essential aspect of hydroxybenzene-derived CPDs, especially for meta-substituted hydroxybenzene, where highly reactive hydrogen atoms at the meta-positions are activated by neighboring electron-donating hydroxyl groups (e.g. *m*-DHB and 1,3,5-THB) (Fig. 7b). The high localized reactivity and co-planarity of substituents enhance the regularity of polycondensation, resulting in CPD particles with unique geometric morphologies, unlike the amorphous polymer clusters formed through random polymerization. Yuan *et al.* prepared triangular CPDs with high color purity and narrow bandwidth emission by utilizing the 3-fold symmetric 1,3,5-THB as the precursor (Fig. 7b, Table S5) [102]. The distinct highly crystallized triangular structure, functionalized with pure electron-donating hydroxyl groups at the edges, exhibited highly delocalized charges and remarkable structural stability, significantly reducing electron–phonon coupling and resulting in high color-purity exciton emissions. Similarly, they later synthesized green- and red-emissive triangular CPDs with narrow full width at half maximum and high PLQY by solvothermal treatment of *m*-DHB (Fig. 7b, Table S5) [103].

The pH also plays a regulatory role in the formation of CPD particles. Moniruzzaman *et al.* developed an easy thermal heating process to synthesize novel shape-specific CPDs (trilateral and quadrilateral) exhibiting multi-color fluorescence emission using 1,3,5-THB as the precursor (Fig. S7a, Table S5) [104]. The controlled bottom-up growth was facilitated by dehydration-mediated covalent polymerization in a sulfuric acid medium. Minervini *et al.* investigated the formation mechanism of *m*-DHB CPDs under varying catalytic conditions, suggesting that both basic and acid catalysts promoted polycondensation reactions via different dehydration processes (Fig. S7b, Table S5) [105]. Acid catalysts yielded rapid carbonization of *m*-DHB, while base catalysts enhanced the PLQY and colloidal stability of the resultant CPDs.

### Polymer precursor-derived CPDs

Polymer precursors suitable for CPD synthesis are typically water-soluble, featuring abundant amino, hydroxy or carboxyl groups (Fig. S8) [106]. These precursors include natural polymers, e.g. chitosan, cellulose and hyaluronic acid (HA), as well as synthetic polymers, e.g. PEI, polyvinyl alcohol (PVA), polyethylene glycol (PEG), polyacrylic acid (PAA) and certain vinyl monomer-derived polymers produced via addition polymerization (Table S6). CPDs derived from these polymer precursors exhibit PL behaviors dominated by the CEE effect, attributed to the exceptional polymeric structures inherited from the precursors [58].

Polymer precursor-derived CPDs synthesized through polycondensation primarily exhibit blue-emissive PL originating from CEE-assisted sub-luminophores, e.g. amide or ester bonds. Polymer skeletons hamper the formation of conjugated molecular-state fluorophores responsible for long-wavelength PL emission, but act as efficient matrices that greatly suppress the non-radiative transitions and disperse sub-luminophores. Regarding the PL mechanism of CEE, sub-luminophores containing electron-rich heteroatoms are immobilized within crosslinked polymer networks, which restrict their vibration and rotation, thereby enhancing PL emission and improving PLQY for CPDs (Fig. S8a). Additionally, the confined domains within CPD particles promote the electron-cloud overlaps and facilitate energy level splitting. These coupled units facilitate the pathway for radiative transitions through enhanced intersystem crossing (Fig. S8b). Benefiting from a prominent CEE effect, polymer precursor-derived CPDs effectively address the issue of solid-state PL quenching, which commonly affects most molecule precursor-derived CPDs, and they display excellent solid-state fluorescence along with unique metal-free RTP (Table S6) [55,107].

Vinyl monomers constitute another primary category of building blocks for constructing CPDs by addition polymerization. Unlike reversible polycondensation, CPDs derived from addition polymerization achieve extremely high product yields, offering significant advantages for practical production. Jiang *et al.* first synthesized CPDs through the direct thermopyrolysis of various 1,4-addition polymers using molecule precursors, allowing precise control over the resulting properties [108]. The obtained CPDs exhibited considerable size uniformity, acceptable PL performance and low cytotoxicity. Xia *et al.* employed a hydrothermal addition polymerization and carbonization strategy to achieve CPDs with ultrahigh yield up to 85% from a single-component acrylamide precursor (Table S6) [109]. They identified the initiator as a critical factor, as no particles were formed in the absence of it. Furthermore, they regulated the RTP lifetime and wavelength by varying the carbonization degree of these CPDs (Table S6), attributing RTP emission to the polymer–carbon hybrid structure with nitrous functional groups serving as molecular-state fluorophore.

Pre-polymerization structural design of polymer precursors enables tailored PL performance and targeted specifications, enhancing the controllability of CPDs. Ge *et al.* synthesized an insoluble polymer precursor (polythiophene phenylpropionic acid, PPA) through oxidative polymerization, subsequently hydrothermally carbonizing the resulting polymers to create water-dispersible CPDs (Fig. S9a) [110]. The red-emissive CPDs, which retained the functional moieties of PPA, demonstrated potential as multi-modal fluorescent, photoacoustic and thermal theranostic agents for cancer diagnosis and treatment in living mice. Tao *et al.* introduced the technique of addition-condensation polymerization to create CPDs with substituents exerting varying degrees of steric hindrance by adjusting methyl group content in co-polymer precursors (Fig. 3i, Fig. S9b) [59]. Results suggested that incorporating methyl units prompted varying degrees of confined-domain CEE, thereby altering energy level distributions and modulating the PL and RTP of CPDs.

Biomass-sourced CPDs demonstrate exceptional performance, along with significant advantages in terms of cost-effectiveness and reduced toxicity. However, these CPDs will not be discussed in detail due to the ambiguous chemical structure of their precursors [111].

## CONCLUSIONS AND OUTLOOK

**Scheme 3.**
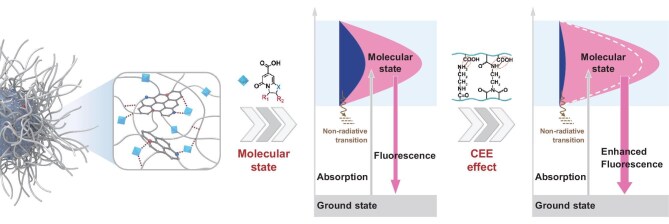
Synergistic PL mechanism arising from molecular-state fluorophores and the CEE effect in CPDs.

In conclusion, we demonstrate the precursor-dependent PL diversity in CPDs from the perspective of luminophores and crosslinking, and elucidate the corresponding effects through examples of several representative CPDs. We emphasize the synergistic PL mechanism of CPDs arising from molecular-state fluorophores and the CEE effect (Scheme 3). Furthermore, CPDs demonstrate properties superior to those of small-molecule or polymeric precursors in many aspects (Table S7), originating from their distinctive nanostructures formed during polymerization and carbonization processes. We expect targeted synthesis of CPDs to fully exploit their luminescent capabilities. Several critical issues merit further exploration and may serve as guidance for future research.

### Scientific classification and definition of CD materials

A robust definition is essential to capture the significant structural and performance differences among CDs synthesized via initial top-down methods compared to later-developed bottom-up methods. Updated classification is always encouraged, informed by the latest insights into particle structures, to address inadequacies in current categorizations. Additionally, distinguishing subcategories of CDs from an experimental perspective, rather than merely a conceptual one, remains an urgent need.

### Purification and identification of the molecular state

Effective purification of CPDs is required to maximize impurity removal while minimizing the depletion of molecular-state fluorophores. NMR is an essential technique for distinguishing molecular-state fluorophores in CPDs from free impurities and for identifying their precise chemical structures [112]. Notably, solvent-sourced PL in CPDs has been successively confirmed, including acetone, DMF, ethanol, ethyl acetate, tetrahydrofuran and diethyl ether. Therefore, the impact of solvents, which may influence the formation of molecular-state fluorophores, warrants further investigation.

### Assessment of CEE-involved emissive behaviors

Crosslinking causes overlaps of electron-cloud and energy level splitting, which enhances electron transfer and leads to unexpected PL emissions in CPDs. To fully understand the contributions of CEE, methodologies for quantifying the degree of crosslinking must be further developed, to supplement the existing empirical conclusions with direct experimental evidence. A comprehensive crosslinked model of CPD particles should be proposed to elucidate essential aspects of the photophysical processes, including CEE contributions to molecular-state fluorophores and effective crosslinking techniques [113,114].

### Theoretical simulations and performance predictions

Advanced computational techniques are poised to provide valuable insights [115]. Molecular dynamics simulations can reflect the collision probability and bonding configurations, potentially reproducing the complex growth processes of CPDs through statistical analysis. Machine learning can correlate the experimental parameters with PL properties of CPDs, thereby streamlining the current trial-and-error approach based on experience and enhancing synthesis efficiency and reproducibility [116]. Fully automated methodologies that integrate machine learning and high-throughput synthesis may facilitate the discovery of CPDs with targeted properties.

### Standardization in CPD synthesis and utilization

Establishing standardized nomenclature is a practical strategy to organize the extensive library of CPD materials. Current guidelines need revision to incorporate consideration of reaction solvents. Furthermore, it is strongly recommended to consult the instituted protocols detailing step-by-step procedures for the synthesis, purification, functionalization and characterization of CPDs [117]. The adoption of emerging techniques and combined methods is encouraged to address remaining challenges regarding the structures of CPDs, aiming for high-quality and large-scale production for diverse applications.

Despite persistent challenges, CPDs exhibit distinct advantages over other luminescent nanomaterials—particularly superior water solubility, easy synthesis and tunable surface chemistry—solidifying their role as luminescent materials over two decades [118]. These attributes drive innovations across diverse applications spanning display technologies, biomedicine, catalytic energy conversion and environmental sensing [119–125]. CPDs demonstrate expanding potential as functional and structural building blocks. The convergence of advances in photophysics, nanomaterial science and characterization techniques will continue to propel transformative developments in this field.

## Supplementary Material

nwaf377_Supplemental_File
