# Supplementary material for: Precursor-dependent photoluminescence diversity in carbonized polymer dots: the luminophores and crosslinking
Source: Natl Sci Rev. 2025 Sep 9;12(10):nwaf377. doi: 10.1093/nsr/nwaf377 (PMC12499410; doi:10.1093/nsr/nwaf377)
Supplement: nwaf377_Supplemental_File [file nwaf377_supplemental_file.pdf]

Supplementary Information for

Precursor-dependent photoluminescence diversity in carbonized polymer dots: the luminophores and crosslinking

Songyuan Tao, Chengyu Zheng and Bai Yang*

State Key Laboratory of Supramolecular Structure and Materials, College of Chemistry, Jilin University, Changchun, 130012, China

***Corresponding author**. E-mail: byangchem@jlu.edu.cn

CONTENTS

Figure S1 Table of contents.

Figure S2 (a) PLQYs of CDs synthesized from different precursors. (b) Nomenclature of CPDs based on the structure of precursors.

Figure S3 (a) The PL mechanism of CEE effect. (b) The CEE effect for sub-luminophores or luminophores: covalent-bond CEE, supramolecular-interaction CEE, ionic-bonding CEE, and confined-domain CEE.

Table S1 Structures and properties of molecular-state fluorophores in CA-amine CPDs.

Figure S4 (a) HETCOR ^{1}H-^{13}C NMR spectra of CA-EDA CPDs. (b) Excitation and emission energies of the studied IPCA-CPDs systems based on multiple snapshots collected from molecular dynamics simulations.

Table S2 Properties of CA CPDs and CA-urea CPDs.

Figure S5 (a) Calculated emission wavelength of GQDs using TDDFT method as a function of the diameter. The solid line represents the linear fit for zigzag-edged GQDs. (b) Calculated UV-vis absorption spectra, HOMO-LUMO gaps, and fluorescence spectra for the low-energy nitrogen-doped and nitrogen-free models. (c) The maximum emission peaks of CA-urea CPDs at different molar ratios of CA to urea and different reaction temperatures.

Table S3 Properties of CPDs synthesized from aminobenzene-based derivatives.

Table S4 Properties of CPDs synthesized from o,m,p-PDA.

Figure S6 (a) Mechanism of the red PL emission: Protonation of surface DAP fluorophore strongly affects the molecular state of CPDs. (b) MALDI-TOF-MASS spectrum and band gap of CPDs (calculated from UV-vis absorption spectra), and the band gap of the oligomers (calculated by theoretic calculation). (c) Core-shell structures and spectral characterization of CPDs.

Table S5 Properties of CPDs synthesized from hydroxybenzene-based derivatives.

Figure S7 (a) Schematic elucidation of the synthesis for shape-specific (trilateral and quadrilateral) multi-fluorescent 1,3,5-THN CPDs. (b) Different mechanisms for base (NaOH) and acid (H_2SO_4) catalyzed condensation of two m-DHB molecules.

Figure S8 (a) Schematic of CEE effect in CPDs. (b) Schematic of the confined-domain CEE effect and its contributions to PL emission.

Table S6 Properties of CPDs synthesized from polymer precursors.

Figure S9 (a) Synthetic route of PPA precursor and CPDs with photothermal effect. (b) Strategy of addition-condensation polymerization to design CPDs with tailored PL performance.

Table S7 Performance comparison among luminescent CPDs, molecules and polymers.

CONTENTS

1. Introduction

2. Evolution of CDs: From fluorescent carbon to CPDs

3. Unique mechanisms of CPDs: Formation and luminescence

3.1. The growth of CPDs: From precursors to luminous nanoparticles

3.2. The dominant PL origin of CPDs: Luminophores and crosslinking

3.2.1. Molecular state originated from organic luminophore

3.2.2. CEE effect originating from crosslinked polymer structure

4. Precursor-dependent PL diversity in CPDs: Reactions and structures

4.1. Non-aromatic molecule precursor-derived CPDs

4.1.1. Citric acid and ethylenediamine

4.1.2. Citric acid and urea

4.2. Aromatic molecule precursor-derived CPDs

4.2.1. Aminobenzene-based derivatives

4.2.2. Hydroxybenzene-based derivatives

4.3. Aromatic molecule precursor-derived CPDs

5. Conclusion and outlook

Figure S1 Table of contents.

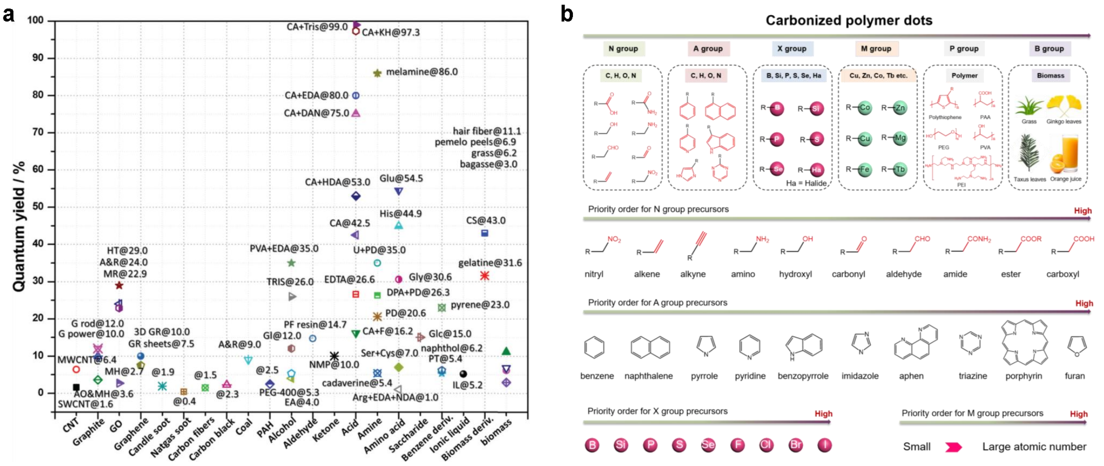

Figure S2 (a) PLQYs of CDs synthesized from different precursors. Reprinted with permission from Ref.[29],copyright 2019, Royal Society of Chemistry. (b) Nomenclature of CPDs based on the structure of precursors. Reprinted with permission from Ref.[33], copyright 2021, Nature Publishing Group.

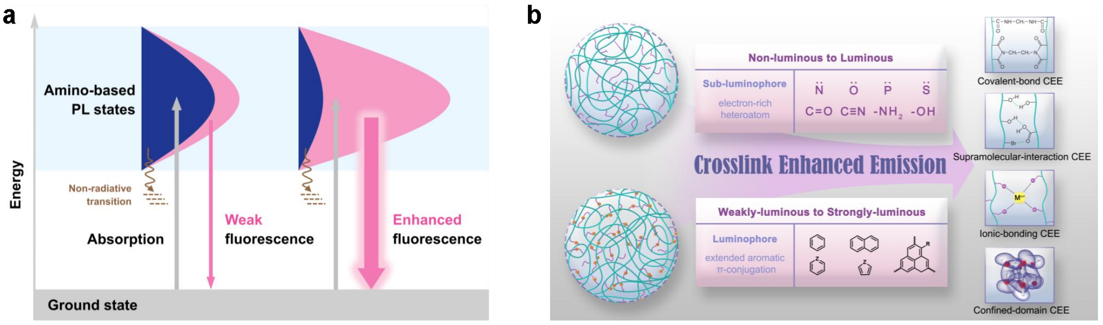

Figure S3 (a) The PL mechanism of CEE effect. Reprinted with permission from Ref.[52], copyright 2014, Royal Society of Chemistry. (b) The CEE effect for sub-luminophores or luminophores: covalent-bond CEE, supramolecular-interaction CEE, ionic-bonding CEE, and confined-domain CEE. Reprinted with permission from Ref.[58], copyright 2020, Wiley-VCH.

Table S1 Structures and properties of molecular-state fluorophores in CA-amine CPDs.

Amine	Fluorophore	QY (%)	EM (nm)	D (nm)	H (nm)	Condition	CPDs name	Ref.
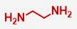	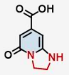	85.84<br>94<br>80.6<br>51<br>60.2	443<br>450<br>445<br>444<br>420~440	2~6<br>2.30<br>2~6<br>3~7<br>4~8	1.26<br>1.0<br>2.81<br>1.7~5.4<br>-	140°C hydrothermal<br>160°C hydrothermal<br>200°C hydrothermal<br>200°C hydrothermal<br>220°C thermal	Citric-acid-ethylenediamine CPDs	40<br>74<br>8<br>126<br>127
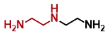	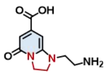	90<br>98	450<br>433	1~3<br>3.1	1.7<br>0.3	180°C reflux<br>180°C hydrothermal	Citric-acid-diethylenetriamine CPDs	128<br>129
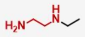	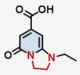	77.07	450	-	-	140°C hydrothermal	Citric-acid-N-ethylethylenediamine CPDs	40
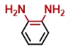	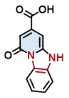	63	450	-	-	180°C pyrolysis	Citric-acid-o-phenylenediamine CPDs	67
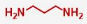	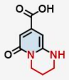	73.55	440	-	-	140°C hydrothermal	Citric-acid-1,3-diaminopropane CPDs	40
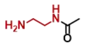	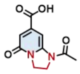	46.36	450	-	-	140°C hydrothermal	Citric-acid-N-acetyletylenediamine CPDs	40
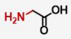	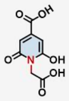	16.9<br>16	415<br>420~440	4~10<br>4~8	-<br>-	200°C thermal<br>220°C thermal	Citric-acid-glycine CPDs	27<br>127
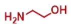	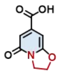	36<br>40.3<br>50<br>75<br>41.9	450<br>455<br>455<br>450<br>420~440	5.13<br>3.5<br>-<br>8.5<br>4~8	1~2<br>-<br>-<br>7<br>-	160°C hydrothermal<br>180°C pyrolysis<br>180°C pyrolysis<br>180°C pyrolysis<br>220°C thermal	Citric-acid-ethanoloamine CPDs	74<br>130<br>21<br>131<br>127
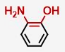	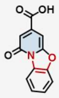	79	418	-	-	180°C pyrolysis	Citric-acid-o-aminophenol CPDs	67
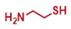	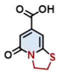	66<br>61<br>66/88	418<br>418<br>415	-<br>-<br>23	-<br>-<br>-	150°C pyrolysis<br>180°C pyrolysis<br>200°C thermal	Citric-acid-cysteamine CPDs	132<br>67<br>133
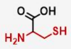	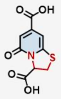	62<br>76<br>70<br>64<br>73<br>70.5	440<br>418<br>420<br>418<br>415<br>420~440	-<br>-<br>-<br>-<br>7<br>4~8	-<br>-<br>-<br>-<br>2<br>-	100°C pyrolysis<br>150°C pyrolysis<br>180°C pyrolysis<br>200°C thermal<br>200°C thermal<br>220°C thermal	Citric-acid-L-cysteine CPDs	66<br>132<br>67<br>132<br>27<br>127
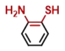	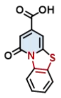	74	450	-	-	180°C pyrolysis	Citric-acid-o-aminothiophenol CPDs	67

Note: (a) EM, D and H represent PL emission wavelength, diameter (as measured by TEM), and height (as measured by AFM) of CPDs. (b) The blue-marked values indicate the QYs of isolated molecules rather than those of the molecular-state fluorophore in CPDs. (c) The pyrolysis approach refers to the direct decomposition of organic precursors during a non-enclosed thermal process. The thermal approach involves heating treatments in a closed reaction vessel, applied to solid-state samples or thick syrup-like samples dissolved in a small amount of solvent. (d) Subsequent tables adhere to the principles outlined above.

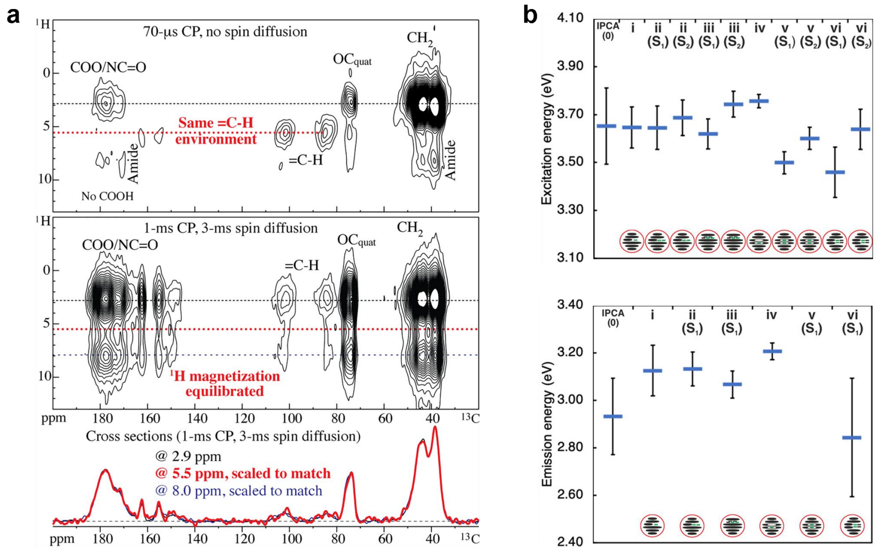

Figure S4 (a) HETCOR ^{1}H-^{13}C NMR spectra of CA-EDA CPDs. Reprinted with permission from Ref.[70], copyright 2019, Wiley-VCH. (b) Excitation and emission energies of the studied IPCA-CPDs systems based on multiple snapshots collected from molecular dynamics simulations. Reprinted with permission from Ref.[49], copyright 2021, Royal Society of Chemistry.

Table S2 Properties of CA CPDs and CA-urea CPDs.

Precursor	Reagent	QY (%)	EM (nm)	D (nm)	H (nm)	Condition	CPDs name	Ref.
**CA**	NaOH<br>-<br>-	22<br>-<br>0.79/0.30	450<br>460<br>450/550	1.8~3.8<br>2~4<br>-	0.5~2.0<br>-<br>-	160°C hydrothermal<br>180°C hydrothermal<br>180°C pyrolysis	Citric-acid CPDs	74<br>134<br>135
**CA**	DMF<br>DMF	18<br>27.5	505<br>645	2.5<br>-	-<br>-	180°C solvothermal<br>180°C solvothermal	Citric-acid CPDs	136<br>134
	formamide<br>formamide<br>formamide	11.9~26.2<br>24<br>22.5	466~637<br>650<br>650	6.8<br>2.4<br>2~4	3<br>-<br>-	160°C microwave<br>180°C solvothermal<br>180°C solvothermal		137<br>136<br>134
	ethanamide	52	605	2.9	-	180°C solvothermal		136
	MeOH	-	580	2~4	-	180°C solvothermal		134
**CA+urea**	-<br>-<br>-<br>-<br>HCl<br>-	58~82<br>44.7<br>32<br>48<br>27.3<br>11	450<br>440<br>448<br>476<br>445<br>435	2.45~7.11<br>5<br>1.7<br>3.2<br>2.81<br>-	-<br>-<br>-<br>-<br>3<br>-	160°C hydrothermal<br>160°C hydrothermal<br>160°C hydrothermal<br>160°C hydrothermal<br>160°C hydrothermal<br>180°C hydrothermal	Citric-acid-urea CPDs	74<br>138<br>75<br>139<br>140<br>135
	H_2O<br>H_2O<br>H_2O<br>H_2O	14<br>-<br>6.4/14.6<br>-	540<br>445<br>540<br>448/420	1~5<br>-<br>-<br>10	1~5<br>-<br>-<br>-	microwave<br>microwave<br>microwave (unsealed)<br>160°C microwave (unsealed)		26<br>72<br>72<br>73
	-<br>-<br>-<br>-<br>-	34<br>84.9/35.9<br>62<br>-<br>-/27	550<br>460/535<br>440<br>450<br>443/514	1.6<br>-<br>-<br>6~10<br>2~7	-<br>-<br>-<br>2~15<br>-	180°C thermal<br>180°C pyrolysis<br>180°C pyrolysis (no O_2)<br>200°C pyrolysis (N_2)<br>pyrolysis (microwave)		136<br>135<br>135<br>44<br>141
**CA+urea**	formamide	4.0~13.3	460~630	2~3	2~3	180°C solvothermal	Citric-acid-urea CPDs	77
	DMF<br>DMF<br>DMF<br>DMF<br>DMF<br>DMF<br>DMF<br>DMF+NaOH<br>DMF+NH_4F	28.7~48.5<br>12.9~52.6<br>60~77<br>8<br>22<br>2.0~27.8<br>29/31<br>46<br>-/9.8	440~640<br>400~670<br>450~550<br>638<br>634<br>444~585<br>445/540<br>580<br>658/772	2~4<br>3.96~4.34<br>2.9~8.3<br>4.5<br>6.7<br>1.7~3.5<br>-<br>4~10<br>2.62	3<br>-<br>3.0~13.4<br>-<br>-<br>-<br>-<br>0.6~2.5<br>-	solvothermal<br>solvothermal<br>160°C solvothermal<br>160°C solvothermal<br>160°C solvothermal<br>180°C solvothermal<br>180°C solvothermal<br>160°C solvothermal<br>180°C solvothermal		142<br>79<br>143<br>75<br>139<br>144<br>135<br>37<br>145
	DMSO	0.2(NIR)	blue~NIR	2~5	0.5~2	160°C solvothermal		146
	DMAC	33	543	4.1	-	160°C solvothermal		139
	EtOH	31.1	513	2.44	3	160°C solvothermal		140
	glycerol	13	550	4.5	-	160°C solvothermal		75
	toluene	28.9	570	2.6	-	200°C solvothermal		147

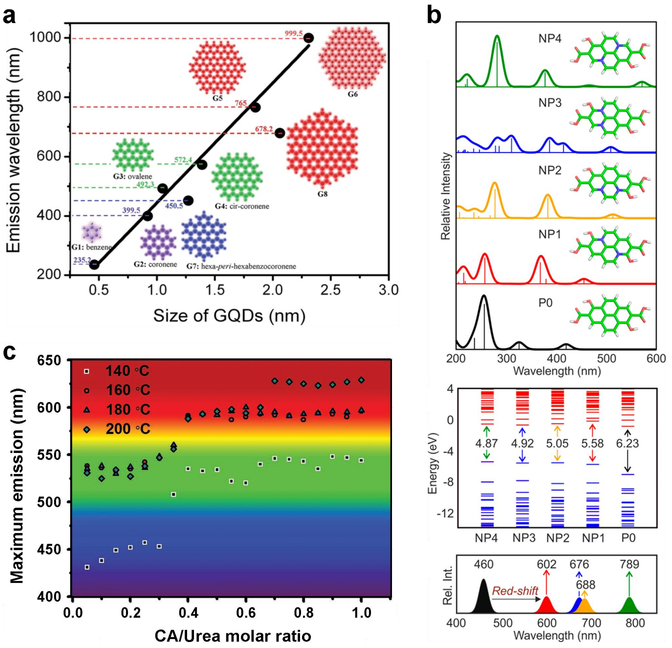

Figure S5 (a) Calculated emission wavelength of GQDs using TDDFT method as a function of the diameter. The solid line represents the linear fit for zigzag-edged GQDs. Reprinted with permission from Ref.[76], copyright 2014, Royal Society of Chemistry. (b) Calculated UV-vis absorption spectra, HOMO-LUMO gaps, and fluorescence spectra for the low-energy nitrogen-doped and nitrogen-free models. Reprinted with permission from Ref.[77], copyright 2017, American Chemical Society. (c) The maximum emission peaks of CA-urea CPDs at different molar ratios of CA to urea and different reaction temperatures. Reprinted with permission from Ref.[79], copyright 2018, Wiley-VCH.

Table S3 Properties of CPDs synthesized from aminobenzene-based derivatives.

Precursor	Reagent	QY (%)	EM (nm)	D (nm)	H (nm)	Condition	CPDs name	Ref.
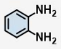	$FeCl_3$<br>-<br>H_3PO_4<br>-<br>$(NH_4)_2S_2O_8$<br>HCl<br>KCl<br>$AlCl_3 \cdot 6H_2O$<br>HNO_3<br>H_3PO_4<br>Na_2SO_4<br>-<br>H_2O<br>H_2SO_4/HCl+oxidant	2.35<br>34<br>15<br>2.15<br>27~61<br>9.1~54.7<br>23~56<br>57<br>10.83<br>17.79<br>14→1.9(H^+)<br>2.0<br>-<br>25.4→8.1(H^+)	550<br>568<br>622<br>573<br>420~590<br>650<br>442~621<br>590~700<br>630/677<br>644/680<br>550→620(H^+)<br>567<br>568<br>606→653(H^+)	3.2<br>2.91<br>2.99<br>6.02<br>1~5<br>~4<br>2.6<br>2.5<br>5.74<br>3.75<br>2<br>4<br>-<br>2.8	-<br>-<br>-<br>-<br>-<br>-<br>4.0~5.5<br>3~4<br>4.1<br>-<br>-<br>-<br>-<br>-	80°C oxidation<br>160°C hydrothermal<br>160°C hydrothermal<br>180°C hydrothermal<br>180°C hydrothermal<br>180°C hydrothermal<br>180~200°C thermal<br>200°C thermal<br>200°C hydrothermal<br>200°C hydrothermal<br>220°C hydrothermal<br>300°C hydrothermal<br>microwave<br>microwave-hydrothermal	o-Phenylenediamine CPDs	148<br>149<br>149<br>150<br>83<br>87<br>151<br>152<br>153<br>86<br>88<br>154<br>155<br>155
	formamide<br>EtOH<br>EtOH<br>EtOH<br>EtOH+$KHSO_4$<br>EtOH<br>EtOH<br>diphenyl ether<br>50%DMF/EtOH<br>10%DMF/EtOH	45<br>3.77<br>10.4(17.6)<br>10.5<br>9.5<br>16.6(26.7)<br>10.0<br>4.8<br>0.8(3.71)<br>3.4(12.39)	533<br>543<br>535<br>541<br>600<br>600/650<br>406<br>505~577<br>410<br>540	3.8<br>8.08<br>8.2<br>2.5<br>2.5<br>3.29<br>2.5<br>~10<br>2.79<br>2.56	-<br>-<br>2~3<br>-<br>-<br>-<br>-<br>~10<br>-<br>-	160°C microwave<br>180°C solvothermal<br>180°C solvothermal<br>180°C solvothermal<br>180°C solvothermal<br>220°C solvothermal<br>230°C solvothermal<br>250°C reflux<br>220°C solvothermal<br>220°C solvothermal	o-Phenylenediamine CPDs	156<br>157<br>28<br>158<br>158<br>90<br>158<br>159<br>90<br>90
	CA+H_2O<br>CA+HCl/H_2SO_4	-<br>24.99~75.41	480~525<br>413~635	5.0<br>2.78~4.05	-<br>-	microwave<br>120~240°C hydrothermal	o-Phenylenediamine-citric acid CPDs	160<br>161
	dopamine+HCl<br>dopamine+H_2SO_4<br>dopamine+H_2SO_4	26.28<br>33.96<br>33.96	710<br>685<br>644/680	7.8<br>5.6<br>3.75	2.1<br>-<br>-	200°C hydrothermal<br>200°C hydrothermal<br>200°C hydrothermal	Dopamine-o-phenylenediamine CPDs	162<br>163<br>86
	catechol+$AlCl_3 \cdot H_2O$	2.65	600/650	10.8	-	200°C thermal	Catechol-o-phenylenediamine CPDs	89
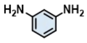	$FeCl_3$<br>-<br>-	3.95<br>7.7~38.3<br>3.30	425<br>512~565<br>420	1.9<br>3.2<br>4.68	-<br>-<br>-	80°C oxidation<br>180°C hydrothermal<br>180°C hydrothermal	m-Phenylenediamine CPDs	148<br>164<br>150
	formamide<br>EtOH<br>EtOH<br>EtOH+H_2SO_4<br>diphenyl ether<br>diphenyl ether	14<br>4.8<br>-<br>43.0<br>9.2~34.5<br>17.6	444<br>435<br>500<br>320/520<br>511~615<br>532~635	4.2<br>6.0<br>~5.0<br>4.3<br>2.6<br><10	-<br>2~3<br><4.5<br>3~4.2<br>2.7<br><10	160°C microwave<br>180°C solvothermal<br>200°C solvothermal<br>200°C solvothermal<br>250°C reflux<br>250°C reflux	m-Phenylenediamine CPDs	156<br>28<br>165<br>165<br>166<br>159
	CA+H_2O	-	565	5.0	-	microwave	m-Phenylenediamine-citric-acid CPDs	160
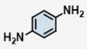	$FeCl_3$<br>-<br>H_3PO_4<br>-<br>-<br>H_2SO_4<br>H_3PO_4<br>HNO_3<br>HF<br>$NH_3 \cdot H_2O$	4.92<br>0.22<br>11.7<br>3.8<br>50.9<br>24.1<br>52.3<br>15.8<br>11.5<br>58.5	600<br>632<br>600<br>400/608<br>519<br>600<br>552<br>600/680<br>600<br>626	2.4<br>6.80<br>-<br>7.0<br>2.57<br>5.07<br>3.51<br>3.46<br>-<br>4.15	-<br>-<br>-<br>-<br>2.28<br>-<br>3.76<br>-<br>-<br>4.15	80°C oxidation<br>180°C hydrothermal<br>180°C hydrothermal<br>200°C hydrothermal<br>200°C hydrothermal<br>200°C hydrothermal<br>200°C hydrothermal<br>200°C hydrothermal<br>200°C hydrothermal<br>200°C hydrothermal	p-Phenylenediamine CPDs	148<br>150<br>167<br>168<br>169<br>85<br>169<br>167<br>167<br>169
	formamide<br>EtOH<br>EtOH+H_2O<br>EtOH<br>DMF<br>cyclohexane<br>toluene<br>diphenyl ether	8<br>20.6(26.1)<br>31.36<br>5.4<br>17.3<br>21.7<br>31.4<br>26.1	574<br>604<br>604<br>400/608<br>400/608<br>400/608<br>400/608<br>538~635	3.7<br>10.0<br>5.02<br>4.0<br>4.0<br>6.0<br>8.0<br>~10	-<br>2~3<br>-<br>-<br>-<br>-<br>-<br>~10	160°C microwave<br>180°C solvothermal<br>180°C solvothermal<br>200°C solvothermal<br>200°C solvothermal<br>200°C solvothermal<br>200°C solvothermal<br>250°C reflux	p-Phenylenediamine CPDs	156<br>28<br>170<br>168<br>168<br>168<br>168<br>159
	urea	6.18~35.14	440~650	2.6	-	160°C hydrothermal	p-Phenylenediamine-urea CPDs	171

Note: H^+ denotes an acidic environment.

Table S4 Properties of CPDs synthesized from aminobenzene-based derivatives.

Precursor	Reagent	QY (%)	EM (nm)	D (nm)	H (nm)	Condition	CPDs name	Ref.
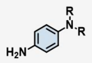	DMF	86.0	637~645	2.2~2.3	1~2	200°C solvothermal	N,N-Dimethyl-p-phenylenediamine CPDs	93
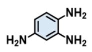	urea	55	585	6.2	-	180°C hydrothermal	1,2,4-Triaminobenzene-urea CPDs	172
	urea+aceton<br>urea+EtOAc<br>urea+THF<br>urea+EtOH	42<br>70<br>79<br>64	486<br>538<br>555<br>566	2.6<br>4.2<br>-<br>-	-<br>-<br>-<br>-	180°C solvothermal<br>180°C solvothermal<br>180°C solvothermal<br>180°C solvothermal	1,2,4-Triaminobenzene-urea CPDs	172<br>172<br>172<br>172
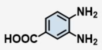	EtOH	24.8	580	3.1	-	200°C solvothermal	3,4-Diaminobenzoic-acid CPDs	173
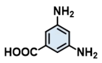	EtOH<br>EtOH+H_3PO_4	29.2<br>69.2	450<br>500	2.05<br>2.65	-<br>-	200°C solvothermal<br>200°C solvothermal	3,5-Diaminobenzoic-acid CPDs	173<br>173
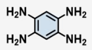	EtOH<br>i-PrOH<br>MeOH<br>n-BuOH<br>DMF	30.2<br>32.7<br>10.0<br>10.0<br>47.6	605<br>600<br>552<br>543<br>527	6.99<br>-<br>-<br>5.32<br>-	-<br>-<br>-<br>-<br>-	170°C hydrothermal<br>170°C hydrothermal<br>170°C hydrothermal<br>170°C hydrothermal<br>170°C hydrothermal	1,2,4,5-Detraaminobenzene CPDs	174<br>174<br>174<br>174<br>174
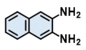	CA+EtOH	73~75	430~513	1.95~2.41	1~2	200°C solvothermal	2,3-Diaminonaphthalene-citric-acid CPDs	92
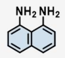	H_2O+EtOH<br>aceton	53.81/13.87<br>69.02/10.11	454<br>546	3.0<br>5.4	2.2~5.4<br>2.2~5.4	180°C solvothermal<br>180°C solvothermal	1,8-Diaminonaphthalene CPDs	175<br>175
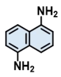	EtOH<br>H_2O+EtOH	73.89/70.05<br>48.8~78.0	405<br>378~597	2.2<br>~9	2.2~5.4<br>5~15	180°C solvothermal<br>180~200°C solvothermal	1,5-Diaminonaphthalene CPDs	175<br>176
	CA+EtOH<br>($NH_3 \cdot H_2O$)<br>CA+EtOH	60~80<br><br>12~58	433<br><br>535~604	2.4<br><br>3.78~6.68	-<br><br>1~2	160°C solvothermal<br>(200°C amination)<br>200°C solvothermal	1,5-Diaminonaphthalene-citric-acid CPDs	94<br>92
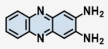	-	>80	400~660	1.8~5.0	<1.0	250~400°C hydrothermal	2,3-Diaminophenazine CPDs	84
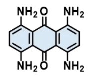	CA	6.8~10.7	700	2.45	0.943	180°C hydrothermal	1,4,5,8-Tetraminoanthraquinone-citric-acid CPDs	95

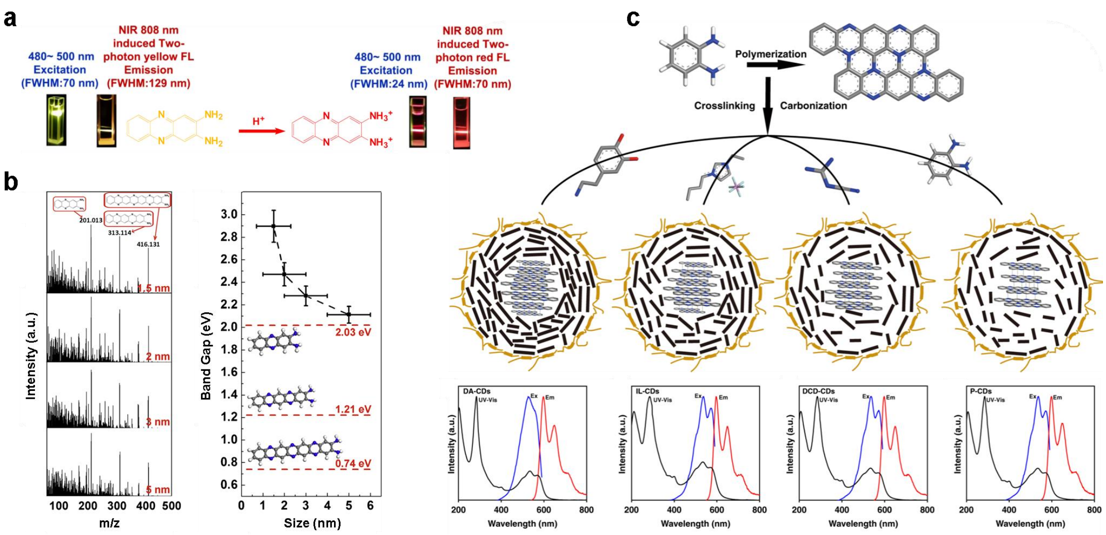

Figure S6 (a) Mechanism of the red PL emission: Protonation of surface DAP fluorophore strongly affects the molecular state of CPDs. Reprinted with permission from Ref.[88], copyright 2022, Nature Publishing Group. (b) MALDI-TOF-MASS spectrum and band gap of CPDs (calculated from UV-vis absorption spectra), and the band gap of the oligomers (calculated by theoretic calculation). Reprinted with permission from Ref.[83], copyright 2014, Elsevier. (c) Core-shell structures and spectral characterization of CPDs. Reprinted with permission from Ref.[86], copyright 2022, Nature Publishing Group.

Table S5 Properties of CPDs synthesized from hydroxybenzene-based derivatives.

Precursor	Reagent	QY (%)	EM (nm)	D (nm)	H (nm)	Condition	CPDs name	Ref.
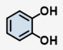	H_2O+H_2SO_4	9.2	360~500	3.5	-	microwave	o-Dihydroxybenzene CPDs	177
	o-PDA	2.65	600/650	10.8	-	100~200°C thermal	o-Dihydroxybenzene-o-phenylenediamine CPDs	89
	N_2H_4+EtOH	3.2~5.3	420~540	5~8	-	160°C solvothermal	o-Dihydroxybenzene-hydrazine-hydrate CPDs	96
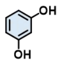	H_2O+H_2SO_4	42.8	350~520	1.9	-	microwave	m-Dihydroxybenzene CPDs	177
	EtOH+oxidant<br>ethylene glycol	81.4<br>42	512<br>520	-<br>1.5	10~15<br>-	180°C solvothermal<br>180°C solvothermal	m-Dihydroxybenzene CPDs	101<br>105
	N_2H_4+EtOH	2.9~4.6	500~540	5~8	-	160°C solvothermal	m-Dihydroxybenzene-hydrazine-hydrate CPDs	96
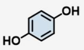	H_2O+H_2SO_4	26.5	400~560	2.8	-	microwave	p-Dihydroxybenzene CPDs	177
	EDA	1.47~78.68	465~550	~5	-	50°C hydrothermal	p-Dihydroxybenzene-ethylenediamine CPDs	99
	EtOH	72~75	520~610	2.5~4.4	-	200°C solvothermal	p-Dihydroxybenzene CPDs	103
	N_2H_4+EtOH	2.6~4.7	417~670	1~9	-	160°C solvothermal	p-Dihydroxybenzene-hydrazine-hydrate CPDs	96
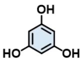	H_2SO_4	1.03~23	438~550	3~8	-	190°C heating	1,3,5-Trihydroxybenzene CPDs	104
	urea+H_2O	5.7~48.2	445~635	2.50~4.95	-	microwave	1,3,5-Trihydroxybenzene-urea CPDs	178
	EtOH<br>EtOH+H_2SO_4	66~72<br>54~62	472~507<br>538~598	1.9~2.4<br>3.0~3.9	-<br>-	200°C solvothermal<br>200°C solvothermal	1,3,5-Trihydroxybenzene CPDs	102<br>102
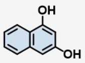	EtOH+KIO_4<br>EtOH+oxidant	53<br>6.6~36.3	628<br>554~625	5.0<br>-	0.5~1.5<br>10~15	180°C solvothermal<br>180°C solvothermal	1,3-Dihydroxynaphthalene CPDs	100<br>101
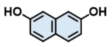	EtOH+oxidant	65.5	388	-	15~20	180°C solvothermal	2,7-Dihydroxynaphthalene CPDs	101

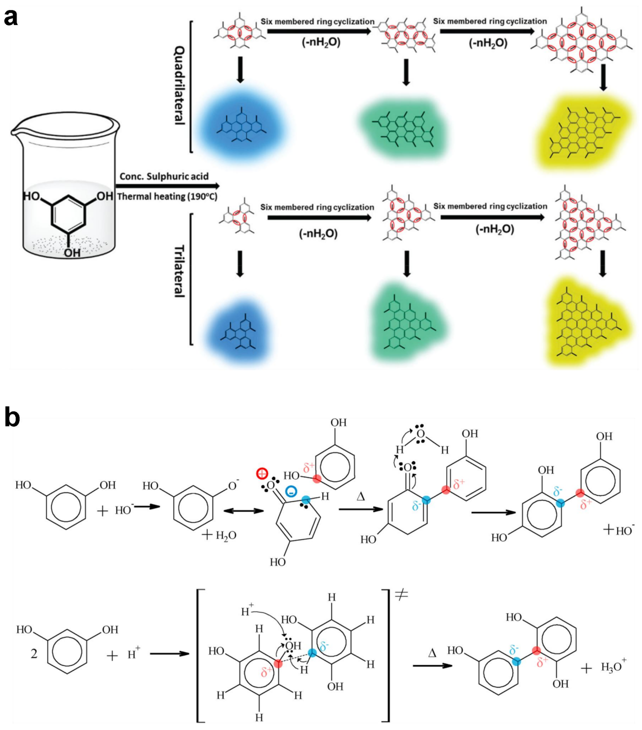

Figure S7 (a) Schematic elucidation of the synthesis for shape-specific (trilateral and quadrilateral) multi-fluorescent 1,3,5-THN CPDs. Reprinted with permission from Ref.[104], copyright 2022, Royal Society of Chemistry. (b) Different mechanisms for base (NaOH) and acid (H_2SO_4) catalyzed condensation of two m-DHB molecules. Reprinted with permission from Ref.[105], copyright 2022, Elsevier.

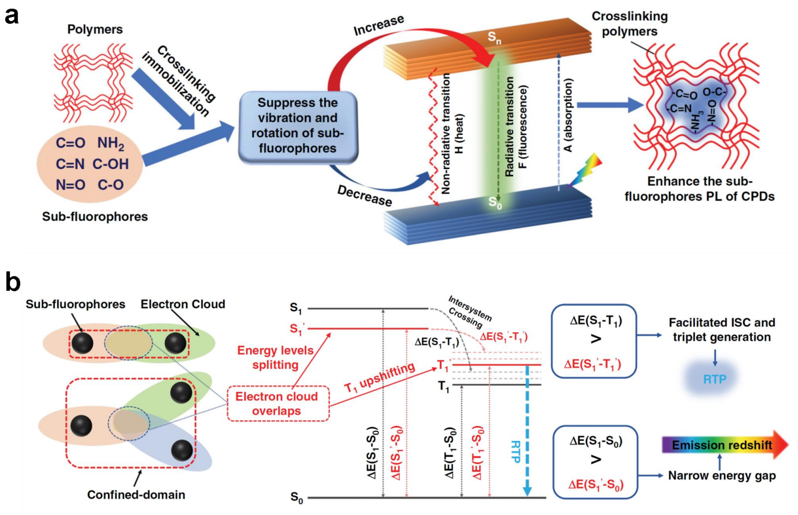

Figure S8 (a) Schematic of CEE effect in CPDs. (b) Schematic of the confined-domain CEE effect and its contributions to PL emission. Reprinted with permission from Ref.[106], copyright 2022, Nature Publishing Group.

Table S6 Properties of CPDs synthesized from polymer precursors.

Polycondensation (Synthetic polymers)									
**Precursor**	**Reagent**	**QY (%)**	**EM (nm)**	**D (nm)**	**H (nm)**	**RTP (nm)**	**Condition**	**CPDs name**	**Ref.**
**PEI**	-	3.06	475	15~100	-	-	CCl_4 crosslinking	Polyethyleneimine CPDs	179
	HNO_3	2	420	12.8	-	-	reflux		180
	$H_2O+H_3PO_4$	11.03	406	1~3	-	512	microwave		181
	CA	48.3	459	4.5	-	-	110°C hydrothermal	Polyethyleneimine-citric-acid CPDs	182
	CA	9.97	440	5.87	-	-	180°C hydrothermal		183
	CA	24.3	455	3.6	-	-	180°C hydrothermal		184
	CA	42	441	4.5~7.5	-	-	180°C hydrothermal		185
	CA	40	430~451	10.5	-	-	180°C reflux		186
**PVA**	-	1.26	475	2~7	-	-	240°C hydrothermal	Polyvinyl-alcohol CPDs	25
	EDA	35	414	9	-	-	200°C hydrothermal	Polyvinyl-alcohol-ethylenediamine CPDs	187
	EDA	13.48	391	2.0	-	-	200°C microwave-hydrothermal		188
	EDA	-	390	>5		546	220°C hydrotherma		189
**PEG**	H_2O	16	445	4.5	-	-	microwave	Polyethylene-glycol CPDs	190
	-	16	460~480	2.0~7.0	-	-	ultrasonic		191
	-	2.51~3.58	450~500	2.3~4.7	1.5~2.5	-	160°C reflux		192
	-	-	450	2.5~4.0	-	-	hydrothermal		193
	-	2.3~3.5	435	3.0~3.5	-	-	120°C hydrothermal		194
	NaOH	19	450	2.5	2~3	-	180°C hydrothermal		195
	P_2O_5	18.4	410	5.5	-	-	200°C thermo-injection		196
	o-PDA	15.2	530~550	3.0	-	-	300°C hydrothermal	Polyethylene-glycol-o-phenylenediamine CPDs	197
**PAA**	H_3PO_4	-	480/565	5.5	-	-	160°C hydrothermal	Polyacrylic-acid CPDs	198
	HCl	0.01~1.27	400~650	3~6	-	550/645	200°C hydrothermal		107
	EDA	28.77	410	5.4	4~6	494	200°C hydrothermal	Polyacrylic-acid-ethylenediamine CPDs	55
	EDA	7.5	~425	3.1	-	520	200°C hydrothermal		199
**Polycondensation (Natural polymers)**									
**Precursor**	**Reagent**	**QY (%)**	**EM (nm)**	**D (nm)**	**H (nm)**	**RTP (nm)**	**Condition**	**CPDs name**	**Ref.**
**Chitosan**	H_2O	6.4	440	4.6	-	-	microwave	Chitosan CPDs	200
	-	43	~460	4~7	5	-	180°C hydrothermal		201
	-	15	422	2.7	1.3	-	180°C microwave-hydrothermal		202
	NaOH	22~27	502	3.8~4.8	1.5~2.1	502	180°C microwave-hydrothermal		202
	-	4.34	390	1~6	1~6	-	300°C pyrolysis (N_2)		203
	EtOH	13.4	~450	8.1	-	-	200°C solvotherma	Chitosan CPDs	204
**Cellulose**	-	1.7	390~530	2~8	7	-	Homogenization	Cellulose CPDs	205
	-	1.0	430~460	1.5	-	-	180°C hydrothermal		206
	urea	21.7	410	4.2	-	-	180°C hydrothermal	Cellulose-urea CPDs	207
**HA**	H_2SO_4	1.9	~420	<20	2.5	-	100°C hydrothermal	Hyaluronic-acid CPDs	208
	-	8.6	454	6.0~9.5	-	-	180°C hydrothermal		209
	$PEI+H_2O$	3.6	470	32.5	3.5	-	microwave	Hyaluronic-acid-polyethyleneimine CPDs	210
	PEI	12.4	470	2.25	-	-	180°C hydrothermal		211
**Addition Polymerization (Vinyl monomers)**									
**Precursor**	**Reagent**	**QY (%)**	**EM (nm)**	**D (nm)**	**H (nm)**	**RTP (nm)**	**Condition**	**CPDs name**	**Ref.**
**Acrylamide**	$K_2S_2O_8$	15.57	380	2.81	-	~500	200°C hydrothermal	Acrylamide CPDs	109
	AIBN	13.88	380	4.56	-	~500	200°C hydrothermal	Acrylamide-N, N'-methylenediacrylamide CPDs	109
	AIBN	14.19	380	4.7	-	485~558	150~300°C hydrothermal		212
	CA+urea	16.5	488	~5	-	~525	200°C hydrothermal	Acrylamide-citric-acid-ethylenediamine CPDs	213

Note: RTP denotes the RTP emission wavelength of CPDs.

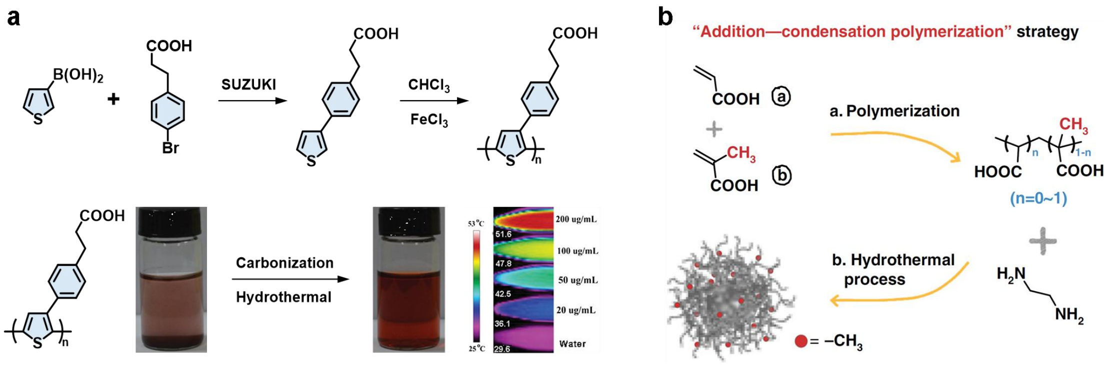

Figure S9 (a) Synthetic route of PPA precursor and CPDs with photothermal effect. Reprinted with permission from Ref.[110], copyright 2015, Wiley-VCH. (b) Strategy of addition-condensation polymerization to design CPDs with tailored PL performance. Reprinted with permission from Ref.[59], copyright 2022, Nature Publishing Group.

Table S7 Performance comparison among luminescent CPDs, molecules and polymers.

Property	CPDs	Molecules	Polymers
Variety	5	5	3
Stability	5	3	4
Processibility	5	2	5
Biocompatibility	5	3	4
Water-solubility	5	2	3
PLQY	4	5	3
FWHM	4	5	3
Atomic-level precision	3	5	3

Note: Performance ranking: 5 (best) → 1 (worst)

REFERENCES

(Continued by the main text)

(126) Kalytchuk S, Zdražil L, Bad'ura Z *et al.* Carbon dots detect water-to-ice phase transition and act as alcohol sensors via fluorescence turn-off/on mechanism. *ACS Nano* 2021; **15**: 6582-93.

(127) Fang Q, Dong Y, Chen Y *et al.* Luminescence origin of carbon based dots obtained from citric acid and amino group-containing molecules. *Carbon* 2017; **118**: 319-26.

(128) Liu X, Li H, Shi L *et al.* Structure and photoluminescence evolution of nanodots during pyrolysis of citric acid: from molecular nanoclusters to carbogenic nanoparticles. *J Mater Chem C* 2017; **5**: 10302-12.

(129) Zhang W, Shi L, Liu Y *et al.* Supramolecular interactions via hydrogen bonding contributing to citric-acid derived carbon dots with high quantum yield and sensitive photoluminescence. *RSC Adv* 2017; **7**: 20345-53.

(130) Hu Y, Yang J, Tian J *et al.* How do nitrogen-doped carbon dots generate from molecular precursors? An investigation of the formation mechanism and a solution-based large-scale synthesis. *J Mater Chem B* 2015; **3**: 5608-14.

(131) Das A, Gude V, Roy D *et al.* On the molecular origin of photoluminescence of nonblinking carbon dot. *J Phys Chem C* 2017; **121**: 9634-41.

(132) Shi L, Yang J, Zeng H *et al.* Carbon dots with high fluorescence quantum yield: the fluorescence originates from organic fluorophores. *Nanoscale* 2016; **8**: 14374-8.

(133) Zhang J, Yang L, Yuan Y *et al.* One-pot gram-scale synthesis of nitrogen and sulfur embedded organic dots with distinctive fluorescence behaviors in free and aggregated states. *Chem Mater* 2016; **28**: 4367-74.

(134) Wei S, Li H, Yin X *et al.* Revealing graphitic nitrogen participating in p–π conjugated domain as emissive center of red carbon dots and applied to red room-temperature phosphorescence. *New J Chem* 2021; **45**: 22335-43.

(135) Otten M, Hildebrandt M, Kühnemuth R *et al.* Pyrolysis and solvothermal synthesis for carbon dots: role of purification and molecular fluorophores. *Langmuir* 2022; **38**: 6148-57.

(136) Wei S, Yin X, Li H *et al.* Multi-color fluorescent carbon dots: graphitized sp^2 conjugated domains and surface state energy level co-modulate band gap rather than size effects. *Chem Eur J* 2020; **26**: 8129-36.

(137) Pan L, Sun S, Zhang A *et al.* Truly fluorescent excitation-dependent carbon dots and their applications in multicolor cellular imaging and multidimensional sensing. *Adv Mater* 2015; **27**: 7782-7.

(138) Li X, Zhang S, Kulinich SA *et al.* Engineering surface states of carbon dots to achieve controllable luminescence for solid-luminescent composites and sensitive Be^{2+} detection. *Sci Rep* 2014; **4**: 4976.

(139) Shen C, Lou Q, Lv C *et al.* Bright and multicolor chemiluminescent carbon nanodots for advanced information encryption. *Adv Sci* 2019; **6**: 1802331.

(140) Yan Y, Xia L, Ma L. Solvent-controlled synthesis of multicolor photoluminescent carbon dots for bioimaging. *RSC Adv* 2019; **9**: 24057-65.

(141) Strauss V, Wang H, Delacroix S *et al.* Carbon nanodots revised: the thermal citric acid/urea reaction. *Chem Sci* 2020; **11**: 8256-66.

(142) Lin S, Chen M, Wang Z *et al.* Construction of full-color light-emitting N-based carbon nanodots and their efficient solid-state materials via tape-casting technology for warm WLED. *Chem Eng J* 2017; **324**: 194-202.

(143) Wang C, He Y, Xu Y *et al.* "Light on" fluorescence carbon dots with intramolecular hydrogen bond-regulated co-planarization for cell imaging and temperature sensing. *J Mater Chem A* 2022; **10**: 2085-95.

(144) Hu T, Wen Z, Wang C *et al.* Temperature-controlled spectral tuning of full-color carbon dots and their strongly fluorescent solid-state polymer composites for light-emitting diodes. *Nanoscale Adv* 2019; **1**: 1413-20.

(145) Jiang L, Ding H, Xu M *et al.* UV–Vis–NIR full-range responsive carbon dots with large multiphoton absorption cross sections and deep-red fluorescence at nucleoli and in vivo. *Small* 2020; **16**: 2000680.

(146) Bao X, Yuan Y, Chen J *et al.* In vivo theranostics with near-infrared-emitting carbon dots—highly efficient photothermal therapy based on passive targeting after intravenous administration. *Light-Sci Appl* 2018; **7**: 91.

(147) Liu Y, Zhang T, Wang R *et al.* A facile and universal strategy for preparation of long wavelength emission carbon dots. *Dalton T* 2017; **46**: 16905-10.

(148) Xia J, Yu Y, Wang J. Fe^{3+}-catalyzed low-temperature preparation of multicolor carbon polymer dots with the capability of distinguishing D_2O from H_2O. *Chem Commun* 2019; **55**: 12467-70.

(149) Zhao J, Li F, Zhang S *et al.* Preparation of N-doped yellow carbon dots and N, P co-doped red carbon dots for bioimaging and photodynamic therapy of tumors. *New J Chem* 2019; **43**: 6332-42.

(150) Cui F, Sun J, de Dieu Habimana J *et al.* Ultrasensitive fluorometric angling determination of staphylococcus aureus in vitro and fluorescence imaging in vivo using carbon dots with full-color emission. *Anal Chem* 2019; **91**: 14681-90.

(151) Ding H, Zhou X, Zhang Z *et al.* Large scale synthesis of full-color emissive carbon dots from a single carbon source by a solvent-free method. *Nano Res* 2021; **15**: 3548-55.

(152) Liu K, Song S, Sui L *et al.* Efficient red/near-infrared-emissive carbon nanodots with multiphoton excited upconversion fluorescence. *Adv Sci* 2019; **6**: 1900766.

(153) Liu J, Li D, Zhang K *et al.* One-step hydrothermal synthesis of nitrogen-doped conjugated carbonized polymer dots with 31% efficient red emission for in vivo imaging. *Small* 2018; **14**: 1703919.

(154) Vedamalai M, Periasamy AP, Wang C *et al.* Carbon nanodots prepared from o-phenylenediamine for sensing of Cu^{2+} ions in cells. *Nanoscale* 2014; **6**: 13119-25.

(155) Ji C, Han Q, Zhou Y *et al.* Phenylenediamine-derived near infrared carbon dots: the kilogram-scale preparation formation process photoluminescence tuning mechanism and application as red phosphors. *Carbon* 2022; **192**: 198-208.

(156) Liu C, Wang R, Wang B *et al.* Orange yellow and blue luminescent carbon dots controlled by surface state for multicolor cellular imaging light emission and illumination. *Microchim Acta* 2018; **185**: 539.

(157) Zhu Y, Bai J, Huang Z *et al.* Study on construction of red carbon nanodots from o-phenylenediamine. *Mater Lett* 2022; **309**: 131397.

(158) An Y, Lin X, Zhou Y *et al.* Red, green and blue light-emitting carbon dots prepared from o-phenylenediamine. *RSC Adv* 2021; **11**: 26915-9.

(159) Sato R, Iso Y, Isobe T. Fluorescence solvatochromism of carbon dot dispersions prepared from phenylenediamine and optimization of red emission. *Langmuir* 2019; **35**: 15257-66.

(160) Pandit S, Mondal S, De M. Surface engineered amphiphilic carbon dots: solvatochromic behavior and applicability as a molecular probe. *J Mater Chem B* 2021; **9**: 1432-40.

(161) Wang B, Yu J, Sui L *et al.* Rational design of multi-color-emissive carbon dots in a single reaction system by hydrothermal. *Adv Sci* 2020; **8**: 2001453.

(162) Lu S, Sui L, Liu J *et al.* Near-infrared photoluminescent polymer–carbon nanodots with two-photon fluorescence. *Adv Mater* 2017; **29**: 1603443.

(163) Wang B, Li J, Tang Z *et al.* Near-infrared emissive carbon dots with 33.96% emission in aqueous solution for cellular sensing and light-emitting diodes. *Sci Bull* 2019; **64**: 1285-92.

(164) Li X, Wang Z, Liu Y *et al.* Bright tricolor ultrabroad-band emission carbon dots for white light-emitting diodes with a 96.5 high color rendering index. *J Mater Chem C* 2020; **8**: 1286-91.

(165) Zhou W, Zhuang J, Li W *et al.* Towards efficient dual-emissive carbon dots through sulfur and nitrogen co-doped. *J Mater Chem C* 2017; **5**: 8014-21.

(166) Wang H, Sun C, Chen X *et al.* Excitation wavelength independent visible color emission of carbon dots. *Nanoscale* 2017; **9**: 1909-15.

(167) Tan C, Su X, Zhou C *et al.* Acid-assisted hydrothermal synthesis of red fluorescent carbon dots for sensitive detection of Fe(III). *RSC Adv* 2017; **7**: 40952-6.

(168) Zhang T, Zhu J, Zhai Y *et al.* A novel mechanism for red emission carbon dots: hydrogen bond dominated molecular states emission. *Nanoscale* 2017; **9**: 13042-51.

(169) Jiao Y, Liu Y, Meng Y *et al.* Novel processing for color-tunable luminescence carbon dots and their advantages in biological systems. *ACS Sustainable Chem Eng* 2020; **8**: 8585-92.

(170) Alaş MÖ, Genç R. Solvatochromic surface-passivated carbon dots for fluorometric moisture sensing in organic solvents. *ACS Applied Nano Materials* 2021; **4**: 7974-87.

(171) Ding H, Yu S, Wei J *et al.* Full-color light-emitting carbon dots with a surface-state-controlled luminescence mechanism. *ACS Nano* 2015; **10**: 484-91.

(172) Kumari R, Sahu SK. Effect of solvent-derived highly luminescent multicolor carbon dots for white-light-emitting diodes and water detection. *Langmuir* 2020; **36**: 5287-95.

(173) Zhao K, Zheng X, Zhang H *et al.* Multi-color fluorescent carbon dots with single wavelength excitation for white light-emitting diodes. *J Alloy Compd* 2019; **793**: 613-9.

(174) Zhao D, Liu X, Zhang Z *et al.* Synthesis of multicolor carbon dots based on solvent control and its application in the detection of crystal violet. *Nanomaterials* 2019; **9**: 1556.

(175) Huo F, Liang W, Tang Y *et al.* Full-color carbon dots with multiple red-emission tuning: on/off sensors in vitro and in vivo multicolor bioimaging. *J Mater Sci* 2019; **54**: 6815-25.

(176) Huo F, Liu Y, Zhu M *et al.* Ultrabright full color carbon dots by fine-tuning crystal morphology controllable synthesis for multicolor bioimaging and sensing. *ACS Appl Mater Interfaces* 2019; **11**: 27259-68.

(177) Wang J, Cheng C, Huang Y *et al.* A facile large-scale microwave synthesis of highly fluorescent carbon dots from benzenediol isomers. *J Mater Chem C* 2014; **2**: 5028-35.

(178) Wang J, Zheng J, Yang Y *et al.* Tunable full-color solid-state fluorescent carbon dots for light emitting diodes. *Carbon* 2022; **190**: 22-31.

(179) Sun B, Zhao B, Wang D *et al.* Fluorescent non-conjugated polymer dots for targeted cell imaging. *Nanoscale* 2016; **8**: 9837-41.

(180) Shen L, Zhang L, Chen M *et al.* The production of pH-sensitive photoluminescent carbon nanoparticles by the carbonization of polyethylenimine and their use for bioimaging. *Carbon* 2013; **55**: 343-9.

(181) Su Q, Yang X. Promoting room temperature phosphorescence through electron transfer from carbon dots to promethazine. *ACS Appl Mater Interfaces* 2021; **13**: 41238-48.

(182) Li J, Liu Y, Shu Q *et al.* One-pot hydrothermal synthesis of carbon dots with efficient up- and down-converted photoluminescence for the sensitive detection of morin in a dual-readout assay. *Langmuir* 2017; **33**: 1043-50.

(183) Jiang B, Yang H, Guo Y *et al.* Developing electropositive citric acid–polyethylenimine carbon quantum dots with high biocompatibility and labeling performance for mesenchymal stem cells in vitro and in vivo. *New J Chem* 2022; **46**: 2508-17.

(184) Wang C, Xu Z, Zhang C. Polyethyleneimine-functionalized fluorescent carbon dots: water stability pH sensing and cellular imaging. *ChemNanoMat* 2015; **1**: 122-7.

(185) Wu X, Wu L, Cao X *et al.* Nitrogen-doped carbon quantum dots for fluorescence detection of Cu^{2+} and electrochemical monitoring of bisphenol A. *RSC Adv* 2018; **8**: 20000-6.

(186) Meierhofer F, Dissinger F, Weigert F *et al.* Citric acid based carbon dots with amine type stabilizers: pH-specific luminescence and quantum yield characteristics. *J Phys Chem C* 2020; **124**: 8894-904.

(187) Chen Y, Zheng M, Xiao Y *et al.* A self-quenching-resistant carbon-dot powder with tunable solid-state fluorescence and construction of dual-fluorescence morphologies for white light-emission. *Adv Mater* 2015; **28**: 312-8.

(188) Zhao L, Li H, Liu H *et al.* Microwave-assisted facile synthesis of polymer dots as a fluorescent probe for detection of cobalt(II) and manganese(II). *Anal BioAnal Chem* 2019; **411**: 2373-81.

(189) Chen Y, He J, Hu C *et al.* Room temperature phosphorescence from moisture-resistant and oxygen-barred carbon dot aggregates. *J Mater Chem C* 2017; **5**: 6243-50.

(190) Jaiswal A, Ghosh SS, Chattopadhyay A. One step synthesis of C-dots by microwave mediated caramelization of poly(ethylene glycol). *Chem Commun* 2012; **48**: 407-9.

(191) Kumar VB, Porat Ze, Gedanken A. Facile one-step sonochemical synthesis of ultrafine and stable fluorescent C-dots. *Ultrason Sonochem* 2016; **28**: 367-75.

(192) Chen M, Wang W, Wu X. One-pot green synthesis of water-soluble carbon nanodots with multicolor photoluminescence from polyethylene glycol. *J Mater Chem B* 2014; **2**: 3937-45.

(193) Jiang Y, Ji C, Wu J *et al.* Formation, photoluminescence and in vitro bioimaging of

polyethylene glycol-derived carbon dots: The molecular weight effects. *Polymer* 2022; **243**: 124625.

(194) Fan R, Sun Q, Zhang L *et al.* Photoluminescent carbon dots directly derived from polyethylene glycol and their application for cellular imaging. *Carbon* 2014; **71**: 87-93.

(195) Wang C, Zhou J, Ran G *et al.* Bi-functional fluorescent polymer dots: a one-step synthesis via controlled hydrothermal treatment and application as probes for the detection of temperature and Fe^{3+}. *J Mater Chem C* 2017; **5**: 434-43.

(196) Li S, Pan R, Ait Mehdi Y *et al.* One-step spontaneous synthesis of fluorescent carbon nanoparticles with thermosensitivity from polyethylene glycol. *New J Chem* 2015; **39**: 7033-9.

(197) Yang P, Ting Y, Gu S *et al.* Effect of solvent on fluorescence emission from polyethylene glycol-coated graphene quantum dots under blue light illumination *Nanomaterials* 2021; **11**: 1383.

(198) Zhao L, Bai Y, Wen Y *et al.* Orange-fluorescence carbon dots employed for the quantitative analysis of silver ions and glyphosine through the off–on mode. *Anal Methods* 2022; **14**: 4230-5.

(199) Li W, Wu S, Xu X *et al.* Carbon dot-silica nanoparticle composites for ultralong lifetime phosphorescence imaging in tissue and cells at room temperature. *Chem Mater* 2019; **31**: 9887-94.

(200) Xiao D, Yuan D, He H *et al.* Microwave-assisted one-step green synthesis of amino-functionalized fluorescent carbon nitride dots from chitosan. *Luminescence* 2013; **28**: 612-5.

(201) Yang Y, Cui J, Zheng M *et al.* One-step synthesis of amino-functionalized fluorescent carbon nanoparticles by hydrothermal carbonization of chitosan. *Chem Commun* 2012; **48**: 380-2.

(202) Wu Q, Wang L, Yan Y *et al.* Chitosan-derived carbon dots with room-temperature phosphorescence and energy storage enhancement properties. *ACS Sustainable Chem Eng* 2022; **10**: 3027-36.

(203) Liu X, Pang J, Xu F *et al.* Simple approach to synthesize amino-functionalized carbon dots by carbonization of chitosan. *Sci Rep* 2016; **6**: 31100.

(204) Briscoe J, Marinovic A, Sevilla M *et al.* Biomass-derived carbon quantum dot sensitizers for solid-state nanostructured solar cells. *Angew Chem Int Ed* 2015; **54**: 4463-8.

(205) Chae A, Choi BR, Choi Y *et al.* Mechanochemical synthesis of fluorescent carbon dots from cellulose powders. *Nanotechnology* 2018; **29**: 165604.

(206) Passos Zattar AP, Paulo de Mesquita J, Pereira FV. Luminescent carbon dots obtained from cellulose and their applications as sensors for metal ions. *Mater Chem Phys* 2022; **290**: 126633.

(207) Shen P, Gao J, Cong J *et al.* Synthesis of cellulose-based carbon dots for bioimaging *ChemistrySelect* 2016; **1**: 1314-7.

(208) Sharker SM, Kim SM, Lee JE *et al.* In situ synthesis of luminescent carbon nanoparticles toward target bioimaging. *Nanoscale* 2015; **7**: 5468-75.

(209) Zhang L, Lin Z, Yu Y *et al.* Multifunctional hyaluronic acid-derived carbon dots for self-targeted imaging-guided photodynamic therapy. *J Mater Chem B* 2018; **6**: 6534-43.

(210) Wang H, Zhang J, Liu Y *et al.* Hyaluronic acid-based carbon dots for efficient gene delivery and cell imaging. *RSC Adv* 2017; **7**: 15613-24.
(211) Zhang M, Zhao X, Fang Z *et al.* Fabrication of HA/PEI-functionalized carbon dots for tumor targeting intracellular imaging and gene delivery. *RSC Adv* 2017; **7**: 3369-75.
(212) Xia C, Zhu S, Zhang S *et al.* Carbonized polymer dots with tunable room-temperature phosphorescence lifetime and wavelength. *ACS Appl Mater Interfaces* 2020; **12**: 38593-601.
(213) Li H, Ye S, Guo J *et al.* The design of room-temperature-phosphorescent carbon dots and their application as a security ink. *J Mater Chem C* 2019; **7**: 10605-12.
